# Small Immunomodulatory Molecules as Potential Therapeutics in Experimental Murine Models of Acute Lung Injury (ALI)/Acute Respiratory Distress Syndrome (ARDS)

**DOI:** 10.3390/ijms22052573

**Published:** 2021-03-04

**Authors:** Dilip Shah, Pragnya Das, Suchismita Acharya, Beamon Agarwal, Dale J. Christensen, Stella M. Robertson, Vineet Bhandari

**Affiliations:** 1 Division of Neonatology, Department of Pediatrics, Drexel University, Philadelphia, PA 19197, USA; shah-dilip@cooperhealth.edu (D.S.); das-pragnya@cooperhealth.edu (P.D.); 2 AyuVis Research, Inc., 1120 South Freeway, Fort Worth, TX 76104, USA; sacharya@ayuvis.com (S.A.); robertson.stella.m@gmail.com (S.M.R.); 3 Pharmacology & Neuroscience, University of North Texas Health Science Center, Fort Worth, TX 76104, USA; 4 GenomeRxUS, Secane, PA 19018, USA; bagarwal@ayuvis.com; 5 Dale J. Christensen Consulting LLC, Cary, NC 27511, USA; dale@djcbio.com; 6 Division of Hematology, Department of Medicine, Duke University Medical Center, Durham, NC 27722, USA; 7 Arrochar Consulting LLC, Fort Worth, TX 76104, USA

**Keywords:** lung inflammation, acute lung injury, pulmonary edema, sepsis, AVR-25, AVR-48

## Abstract

Background: Acute lung injury (ALI) or its most advanced form, acute respiratory distress syndrome (ARDS) is a severe inflammatory pulmonary process triggered by a variety of insults including sepsis, viral or bacterial pneumonia, and mechanical ventilator-induced trauma. Currently, there are no effective therapies available for ARDS. We have recently reported that a novel small molecule AVR-25 derived from chitin molecule (a long-chain polymer of N-acetylglucosamine) showed anti-inflammatory effects in the lungs. The goal of this study was to determine the efficacy of two chitin-derived compounds, AVR-25 and AVR-48, in multiple mouse models of ALI/ARDS. We further determined the safety and pharmacokinetic (PK) profile of the lead compound AVR-48 in rats. Methods: ALI in mice was induced by intratracheal instillation of a single dose of lipopolysaccharide (LPS; 100 µg) for 24 h or exposed to hyperoxia (100% oxygen) for 48 h or undergoing cecal ligation and puncture (CLP) procedure and observation for 10 days. Results: Both chitin derivatives, AVR-25 and AVR-48, showed decreased neutrophil recruitment and reduced inflammation in the lungs of ALI mice. Further, AVR-25 and AVR-48 mediated diminished lung inflammation was associated with reduced expression of lung adhesion molecules with improvement in pulmonary endothelial barrier function, pulmonary edema, and lung injury. Consistent with these results, CLP-induced sepsis mice treated with AVR-48 showed a significant increase in survival of the mice (80%) and improved lung histopathology in the treated CLP group. AVR-48, the lead chitin derivative compound, demonstrated a good safety profile. Conclusion: Both AVR-25 and AVR-48 demonstrate the potential to be developed as therapeutic agents to treat ALI/ARDS.

## 1. Introduction

Acute lung injury (ALI) and acute respiratory distress syndrome (ARDS) are associated with a variety of insults, including massive hemorrhage, systemic bacterial or viral infection, inhalation of noxious agents, burns, and blast trauma, and have an overall mortality of 30–40% [1]. ARDS is characterized by acute inflammation, microvascular damage, and increased pulmonary vascular and epithelial permeability, frequently resulting in acute respiratory failure and death [2,3,4]. It is estimated that >200,000 individuals develop ALI and ARDS each year in the United States, resulting in nearly 75,000 deaths annually [3,5]. The recent COVID-19 pandemic also witnessed a huge number of severe ARDS cases, which impacted the health sector, globally. To date, effective pharmacological therapies have not been identified for ARDS and only ventilation strategies with low tidal volume adjustments have been shown to reduce mortality in ARDS patients [6]. Early administration of corticosteroids to patients with sepsis, who are more likely to develop ARDS, also does not prevent the development of this dreadful disease [7]. Numerous pharmacologic therapies, including the use of inhaled or instilled synthetic surfactant, intravenous (IV) antibody to endotoxin, ketoconazole, and ibuprofen [8] have been tried, but have proved to be ineffective [9]. Statins, which appeared promising in small studies, also did not show benefit in a recently published randomized trial with 1636 patients with ALI [9]. Inhaled nitric oxide (iNO), a potent pulmonary vasodilator, seemed promising in early trials, but in larger controlled trials, it did not change mortality rates in adults with ARDS [10]. Hence, there is a clear unmet need for preventive as well as therapeutic approaches for ALI and ARDS.

Earlier studies demonstrated that low molecular weight chitosan, such as chitohexaose, modulate macrophages via alternate activation to a non-inflammatory phenotype by interacting with the host immune receptor toll-like receptor (TLR) 4, producing interleukin (IL)-10 [11]. In doing so, it blocks lipopolysaccharide (LPS)-induced inflammatory mediators such as tumor necrosis factor (TNF)-α and IL-6 in vitro (both in murine macrophages and human peripheral blood monocytes-hPBMC) and reduces endotoxemia, in vivo [11]. The functions of alternatively activated macrophages involve the control of inflammatory responses, enhanced phagocytic activity and tissue repair [12,13].

The small molecule immunomodulator AVR-48 [14] demonstrates an increased IL-10 expression with or without LPS and decreases TNF-α in an in vitro hPBMC model, similar to our earlier reported chitohexaose analog AVR-25 [15], which demonstrated significant anti-inflammatory activities and mitigated cecal ligation and puncture (CLP)-induced sepsis and lung injury in both adult and aged mice. The safety profile of AVR-25 has been published [15]. The goal of this study was to determine the efficacy of two chitin derivative compounds, AVR-25 and AVR-48, in multiple mouse models of ALI/ARDS. We further determined the safety and pharmacokinetic (PK) profile of the lead compound AVR-48 in rats. We report, here, the safety and PK profiling of compound AVR-48 to determine the maximum tolerated dose (MTD) and drug concentration in plasma and bronchoalveolar lavage fluid (BALF) in rats, as we wanted to use this compound as a candidate drug for ARDS. Both compounds AVR-25 and AVR-48 were studied in the LPS and hyperoxia-induced experimental ARDS in mice. Our studies show that the treatment of LPS or hyperoxia-induced ALI mice with either AVR-25 or AVR-48 results in decreased lung inflammation, cell death, and improved pulmonary endothelial barrier function with overall recovery in pulmonary edema and injury. 

We also tested AVR-48 in the CLP-induced mouse model of sepsis, since sepsis is another cause of ALI and ARDS that is associated with disseminated intravascular coagulation due to multi-organ failure and increases the risk of progression to ALI/ARDS [16]. Compound AVR-48 was able to decrease mortality and overall injury of all organs (including the lung) in the CLP model. These data suggest that AVR-25 or AVR-48 can be used as potential therapeutic agents for treating ALI/ARDS.

## 2. Results

### 2.1. Structure of AVR-48

**Figure 1:**
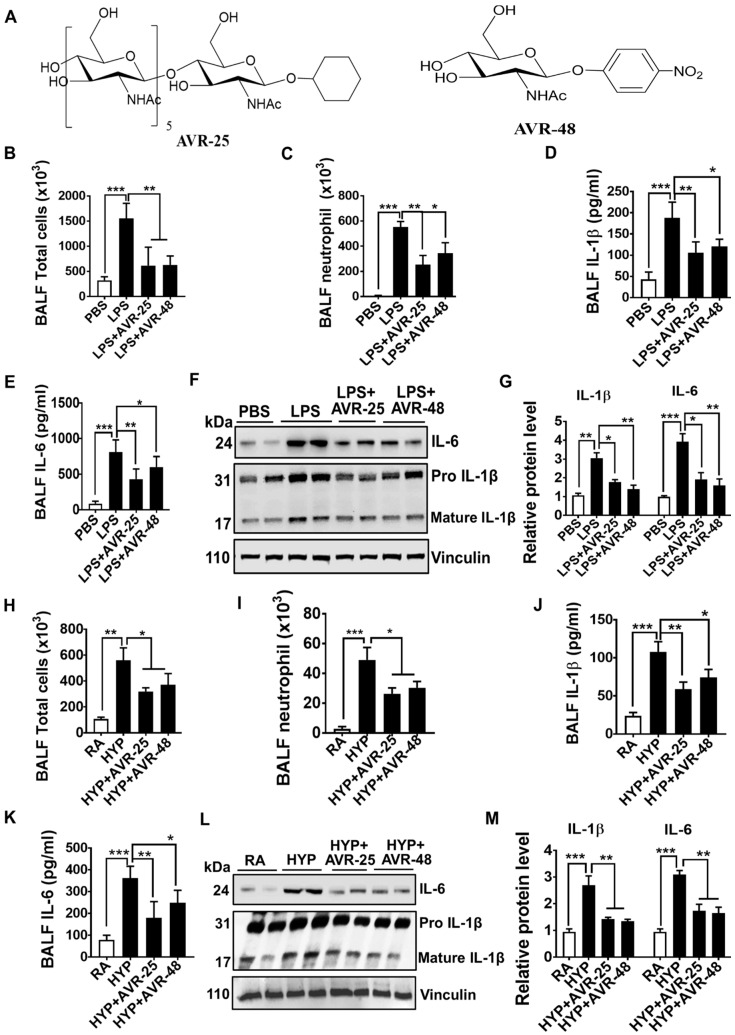
AVR-25 or AVR-48 treatment of LPS- and hyperoxia-induced ALI mice ameliorated lung inflammation. (**A**) Chemical structure of the discussed compounds: Compound AVR-48 is a short chain analog of our previously published compound AVR-25 with IUPAC name as N-((2S,3R,4R,5S,6R)-4,5-dihydroxy-6-(hydroxymethyl)-2-(4-nitrophenoxy)tetrahydro-2H-pyran-3-yl) acetamide. (**B**,**C**,**H**,**I**) Total inflammatory and neutrophil cell counts in BALF of LPS- and hyperoxia-induced ALI in mice treated with or without AVR-25 or AVR-48 (*n* = 5–6, * *p* < 0.05, ** *p* < 0.01 and *** *p* < 0.001). (**D**,**E**,**J**,**K**) ELISA assay for IL-1β and IL-6 in BALF of LPS- and hyperoxia-induced ALI in mice treated with or without AVR-25 or AVR-48 (*n* = 5–6, * *p* < 0.05, ** *p* < 0.01 and *** *p* < 0.001). (**F**,**G**,**L**,**M**) Western Blots analysis for IL-1β and IL-6 in the lungs of LPS- and hyperoxia-induced ALI in mice treated with or without AVR-25 or AVR-48. Right panel shows densitometric quantification of the immunoblots (*n* = 5–6, * *p* < 0.05, ** *p* < 0.01 and *** *p* < 0.001). Data are expressed as mean ± SEM. For statistical analysis, student’s unpaired *t*-test and one-way analysis of variance (ANOVA) followed by Tukey post hoc analysis were used. LPS: lipopolysaccharide; ALI: acute lung injury; BALF: bronchoalveolar lavage fluid; IL: interleukin; PBS: phosphate buffered saline; RA: room air; HYP: hyperoxia.

AVR-48 is a shorter sugar chain analog of AVR-25 and contains only one N-acetyl-glucosamine moiety (Figure 1A) with a beta anomeric substitution of *p*-nitro phenol. Compound AVR-25 has six N-acetyl glucosamine moieties linked to one another by β 1→4 linkage and with a beta anomeric substitution of cyclohexyl group. The structure of AVR-25 has been published previously [15].

### 2.2. Safety Profile of AVR-48 in Adult Rats

To assess the safety of AVR-48, two doses of IV bolus injections were given to both male and female rats, 6–7 h apart, at a total daily dose of up to 100 mg/kg/day. At 100 mg/kg clinical signs of hypoactivity, respiratory changes, changes in posture, and loss of motor coordination were observed. A single animal that received a total daily dose of 100 mg/kg died. No adverse signs were observed at the lower dose of 75 mg/kg/day. Repeat administration of AVR-48 at doses of 40 and 80 mg/kg/day, when administered as two daily doses of 20 and 40 mg/kg for three consecutive days was well tolerated with no adverse clinical signs. There were no changes in any of the hematology or clinical chemistry parameters that could be attributed to the IV dosing of AVR-48 for three consecutive days at doses up to 80 mg/kg/day. Signs of discoloration, swelling, and macroscopic and microscopic signs of local irritation occurred at the site of administration in all treatment groups and were attributed to the administration vehicle (formulation of 10% DMSO, 20% Tetraglycol, and 20% PEG 400 in sterile water). Further, there was no evidence of any AVR-48 related systemic gross observations at necropsy and no adverse findings attributable to AVR-48. Based on parameters monitored in this study, the MTD and no observable adverse effect level (NOAEL) were considered to be 80 mg/kg/day.

### 2.3. PK Profile of AVR-48 in Rat Plasma and BALF

We developed an in-house HPLC method to study the PK profile in plasma and BALF after AVR-48 dosing. Blood samples were collected, and plasma prepared following the first and last doses in the repeat dose study. Maximum plasma concentration (T_max_) was measured immediately after dosing. T_max_ of AVR-48 was generally followed by a biexponential decline of AVR-48 levels (Figure S1), with the C_max_ of 363.7 ± 9.36 µM at 40 mg/kg dose and 749.34 ± 5.25 µM at 80 mg/kg dose (2-fold) where the half-life (T_1/2_) was estimated (when estimable) between 1.40 and 2.34 h at both doses. Quantitation of AVR-48 in BALF demonstrated measurable concentrations at 6 h post administration and levels below the limit of detection by 12 h. At the 6 h time point, higher exposure of AVR-48 in BALF relative to plasma was observed, with a BALF-to-plasma ratio ranging between 2.81 and 9.08. The toxicokinetic parameters at day 1 and day 3 are presented in the Tables S1 and S2. 

In our earlier report [15], we demonstrated the efficacy of AVR-25 in the CLP mouse model at the 10 mg/kg IV dose. Here, the plasma C_max_ values at 40 mg/kg provided us the guidance that, a single IV or intra-peritoneal (IP) injection of 10 mg/kg dose of AVR-48 will provide C_max_ of ~91 µM in plasma sufficient to produce the desired anti-inflammatory therapeutic effect in the mouse. So, we selected a 10 mg/kg dose for both compounds AVR-25 and AVR-48 in the mouse lung injury studies. 

Other PK parameters were also determined. The volume of distribution (V_d_) and clearance (CL) were estimated between 1510 and 3720 mL/kg and between 745 and 1100 mL/h/kg, at day 1 and day 3 of dosing, respectively. In general, the area under curve, AUC_(0-t)_ increased in a dose-dependent manner between 40 and 80 mg/kg/day dose groups (Tables S1 and S2). The exposure to AVR-48 on day 3 did not change substantially after three days of twice daily administration. There was no accumulation of the compound. 

### 2.4. Effect of AVR-25 and AVR-48 on Pro-Inflammatory Cytokines in the Lung

To study the effect of AVR-25 and AVR-48 on lung inflammation in ALI mice, we measured total inflammatory cells, neutrophil cells, and selected pro-inflammatory cytokines in BALF. As shown in Figure 1B–G, LPS-induced ALI mice treated with two doses of AVR-25 or AVR-48 (10 mg/kg body weight administered after 4 h and 12 h of LPS instillation) attenuated total immune cells, as well as neutrophil cell counts as demonstrated by decreased counts of the total inflammatory (Figure 1B) and neutrophil cells (Figure 1C) in the BALF. Consistent with decreased neutrophil counts, pro-inflammatory cytokines IL-1β and IL-6 levels were also decreased in BALF as well as lung tissues of ALI mice treated with AVR-25 or AVR-48 (Figure 1D–G). To further strengthen our hypothesis that AVR-25 and AVR-48 diminish lung inflammation in ALI mice, we next measured lung inflammation markers in the hyperoxia-induced model of ALI. Similar to the anti-inflammatory effect shown by AVR-25 and AVR-48 in the LPS-induced murine model of ALI, these molecules also diminished the total immune cells and neutrophils infiltration into the lung (Figure 1H,I). This diminished recruitment of inflammatory cells was also associated with decreased levels of pro-inflammatory cytokines in BALF as well as in lung tissues of ALI mice treated with AVR-25 or AVR-48 as compared to untreated ALI mice (Figure 1J–M). Altogether, these data suggest that AVR-25 and AVR-48 attenuate lung inflammation induced by LPS and hyperoxia exposure. 

### 2.5. Effect of AVR-25 and AVR-48 on the Anti-Inflammatory Cytokine IL-10 in BALF

**Figure 2:**
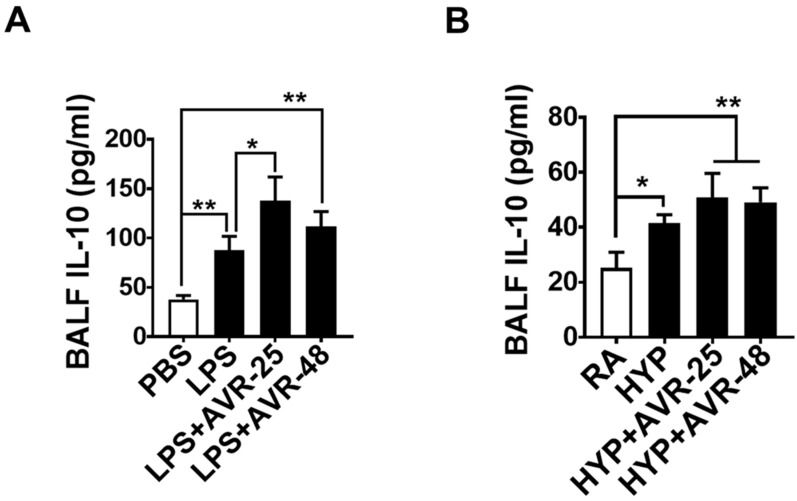
AVR-25 or AVR-48 treatment enhances anti-inflammatory cytokine in LPS- and hyperoxia-induced ALI mice. (**A**,**B**) ELISA assay for the levels of anti-inflammatory cytokine IL-10 in the BALF (*n* = 5–6, * *p* < 0.05 and ** *p* < 0.01). Data are expressed as mean ± SEM. For statistical analysis, student’s unpaired *t*-test and one-way analysis of variance (ANOVA) followed by Tukey post hoc analysis were used. LPS: lipopolysaccharide; ALI: acute lung injury; BALF: bronchoalveolar lavage fluid; IL: interleukin; PBS: phosphate buffered saline; RA: room air; HYP: hyperoxia.

Decreased IL-10 has been reported in clinical specimens such as plasma and BALF from ARDS patients [17], and the treatment of ALI mice with recombinant IL-10/Fc [18] or an IL-10 stimulator molecule S100A8 has been associated with decreased lung inflammation and lung injury [19,20]. Hence, we investigated whether the decreased pro-inflammatory cytokines in the AVR-25 or AVR-48 treated animals were also associated with increased production of IL-10 in the BALF. Interestingly, we found that LPS as well as hyperoxia exposure led to increased levels of IL-10 in the BALF; and, after treatment with AVR-25 or AVR-48, there was a further increase in the levels of IL-10 as compared to non-treated ALI mice (Figure 2A,B). These results indicate that both AVR-25 and AVR-48 have an anti-inflammatory effect in the lung following the insult, as reflected by an increase of IL-10, after treatment.

### 2.6. Effect of AVR-25 and AVR-48 on Pulmonary Vascular Leakage in ALI Mice

**Figure 3:**
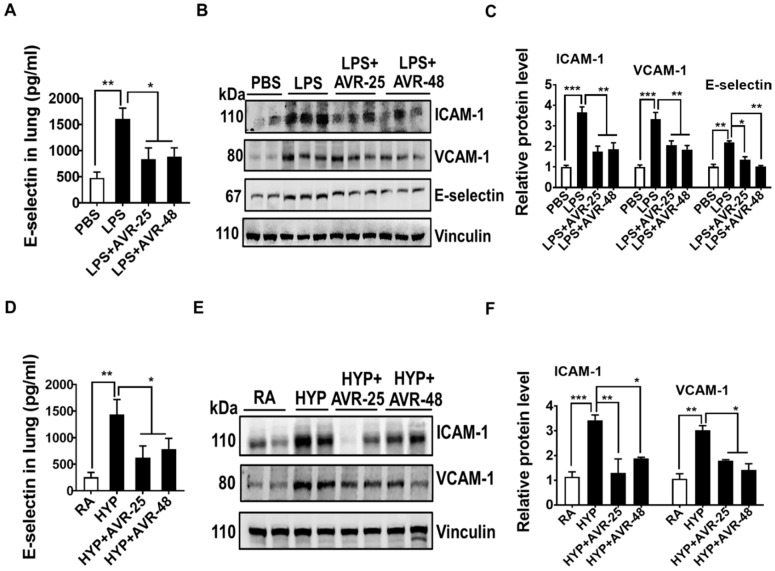
AVR-25 or AVR-48 treatment suppresses the production of lung adhesion molecules in in LPS- and hyperoxia-induced ALI mice. (**A**,**D**) ELISA assay for the levels of E-selectin in lungs of LPS- and hyperoxia-induced ALI in mice treated with or without AVR-25 or AVR-48 (*n* = 5–6, * *p* < 0.05 and ** *p* < 0.01). (**B**,**C**,**E**,**F**) Western Blots analysis for ICAM-1, VCAM-1, and E-selectin in the lungs of LPS- and hyperoxia-induced ALI in mice treated with or without AVR-25 or AVR-48. Right panel shows densitometric quantification of the immunoblots (*n* = 4–6, * *p* < 0.05, ** *p* < 0.01 and *** *p* < 0.001). Data are expressed as mean ± SEM. For statistical analysis, student’s unpaired *t*-test and one-way analysis of variance (ANOVA) followed by Tukey post hoc analysis were used. LPS: lipopolysaccharide; ALI: acute lung injury; PBS: phosphate buffered saline; RA: room air; HYP: hyperoxia.

To assess whether the diminished lung inflammation mediated by AVR-25 and AVR-48 in ALI also impacted the production of lung adhesion molecules, endothelial adherens junction (AJ) proteins, and pulmonary edema, the expression of lung adhesion molecules intercellular adhesion molecule (ICAM)-1, vascular cell adhesion molecule (VCAM)-1, and E-selectin, were measured in whole lung tissues. Similarly, lung endothelial AJ proteins, vascular endothelial (VE)-cadherin and β-catenin, were analyzed in whole lung tissues and levels of total protein and Evans Blue dye were measured in BALF and lung tissues in these mice. In both LPS and hyperoxia-induced ALI mice treated with AVR-25 or AVR-48, there was decreased expression of lung adhesion molecules ICAM-1, VCAM-1, and E-selectin in whole lung tissue (Figure 3A–F). 

**Figure 4:**
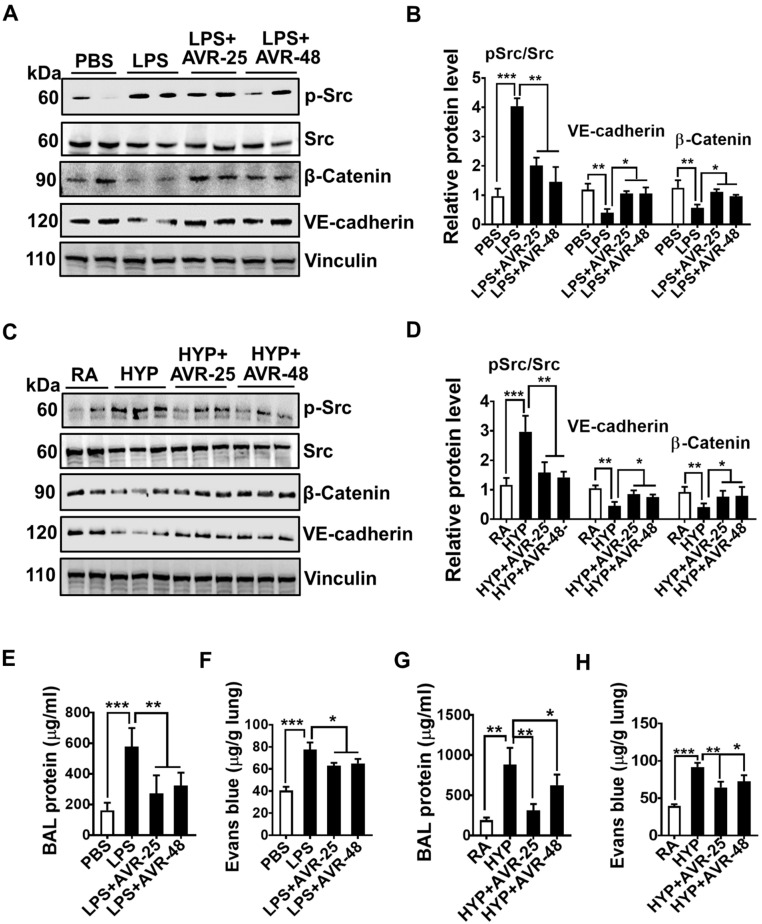
AVR-25 or AVR-48 treatment to LPS- and hyperoxia-induced ALI mice showed an improved endothelial barrier function. (**A**,**C**) Western blotting showing expression of junctional adherence proteins VE-cadherin, β-catenin, and Src in lungs of LPS- and hyperoxia-induced lung injury in mice treated with or without AVR-25 or AVR-48. (**B**,**D**) Densitometric quantification of the immunoblots (*n* = 4–6, * *p* < 0.05 ** *p* < 0.01 and *** *p* < 0.001). (**E**–**H**) Pulmonary edema as measured by total protein concentration in the BALF and Evans Blue dye concentration in the lungs of LPS- and hyperoxia-induced lung injured mice (*n* = 5–6, * *p* < 0.05 ** *p* < 0.01 and *** *p* < 0.001). Data are expressed as mean ± SEM. For statistical analysis, student’s unpaired *t*-test and one-way analysis of variance (ANOVA) followed by Tukey post hoc analysis were used. LPS: lipopolysaccharide; ALI: acute lung injury; BAL: bronchoalveolar lavage fluid; PBS: phosphate buffered saline; RA: room air; HYP: hyperoxia.

Consistent with decreased expression of lung adhesion molecules, there were increased expression of endothelial AJ proteins, vascular endothelial (VE)-cadherin, and β-catenin, along with decreased expression of phosphorylated-Src in whole lungs of ALI-induced by LPS and hyperoxia (Figure 4A–D). AVR-25 and AVR-48 mediated improved endothelial barrier function is also supported by decreased pulmonary edema as demonstrated by diminished levels of total proteins in BALF (Figure 4E,G) and Evans Blue dye leak concentration (Figure 4F,H) in the lungs. These data suggest that AVR-25 and AVR-48 are able to improve pulmonary vascular injury in mouse models of ALI. 

### 2.7. Effect of AVR-25 and AVR-48 on Lung Cell Death in ALI Mice

**Figure 5:**
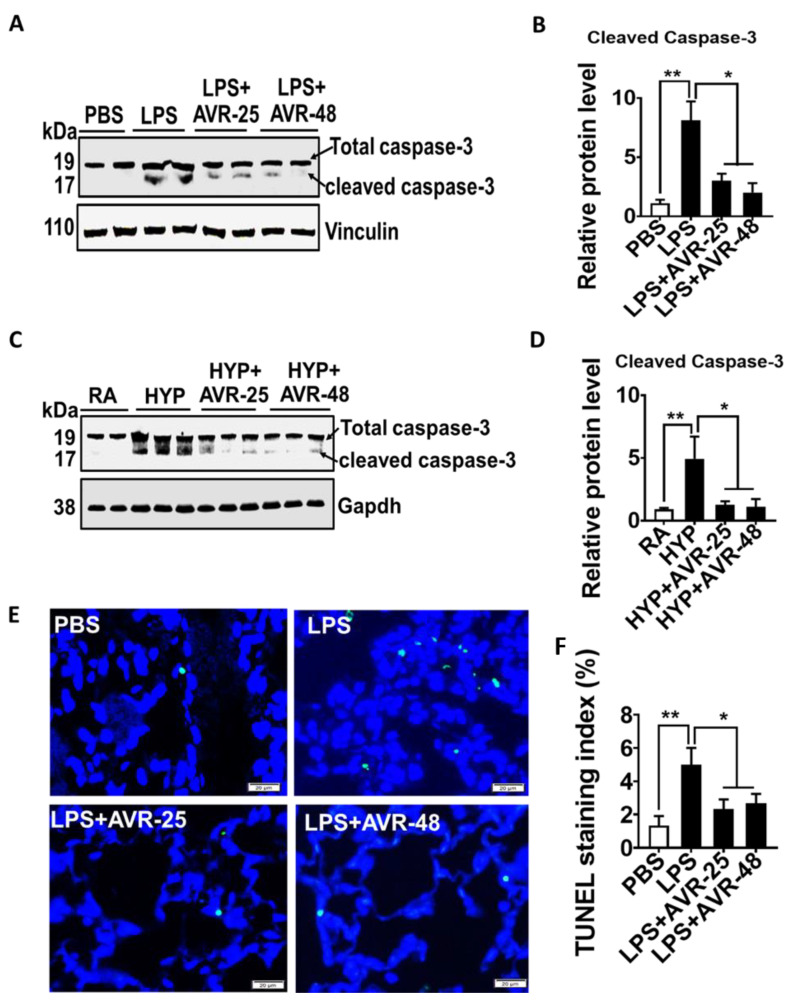
AVR-25 or AVR-48 treatment attenuates lung cell death in LPS- and hyperoxia-induced ALI mice. (**A**–**D**) Western blot analysis for cleaved caspase-3 in lungs of LPS and hyperoxia-induced lung injury in mice treated with or without AVR-25 or AVR-48. Densitometric quantification of the immunoblots is shown right to immunoblots (*n* = 4–6, * *p* < 0.05 and ** *p* < 0.01). (**E**,**F**) Representative figure of TUNEL staining (green color) of apoptotic cells and quantification of apoptotic cells in lungs of LPS and hyperoxia-induced lung injury in mice treated with or without AVR-25 or AVR-48 (* *p* ≤ 0.05). Scale bar = 20 µm. Data are expressed as mean ± SEM. For statistical analysis, student’s unpaired *t*-test and one-way analysis of variance (ANOVA) followed by Tukey post hoc analysis were used. LPS: lipopolysaccharide; ALI: acute lung injury; PBS: phosphate buffered saline; RA: room air; HYP: hyperoxia.

We further analyzed whether AVR-25 and AVR-48 treatment also influences overall lung cell death in ALI mice. As shown in Figure 5A–D, ALI mice treated with AVR-25 or AVR-48 showed decreased lung cell death as manifested by reduced expression of cleaved caspase-3. These results were further supported by decreased TUNEL staining in the lung sections of ALI mice treated with AVR-25 (Figure 5E,F). These results suggest that AVR-25 and AVR-48 are able to attenuate LPS or hyperoxia induced cell death in the lung cells. 

### 2.8. Effect of AVR-25 and AVR-48 on LPS-and Hyperoxia-Induced Lung Histopathology

**Figure 6:**
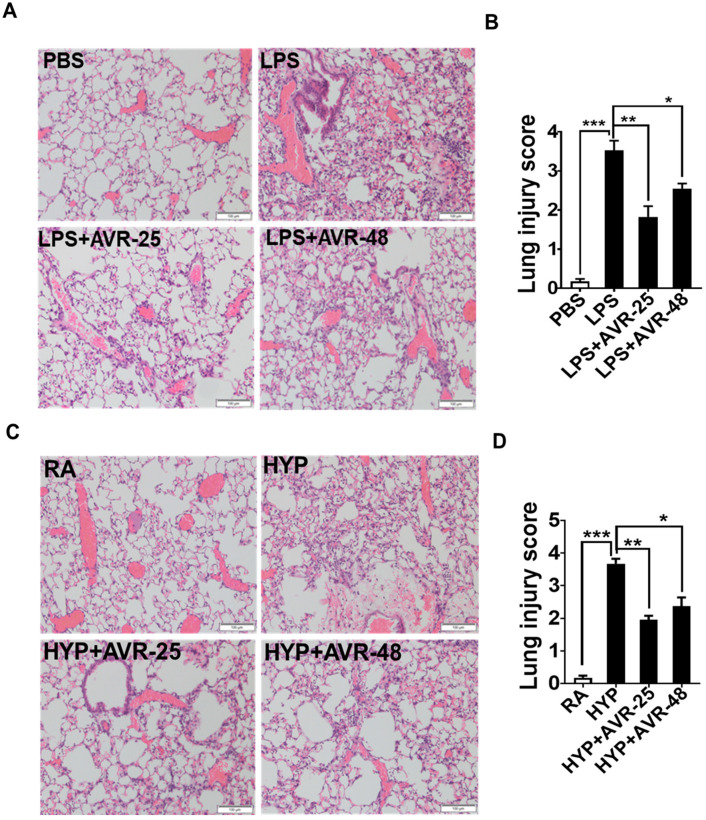
AVR-25 or AVR-48 treatment attenuated pulmonary injury in LPS- and hyperoxia-induced ALI mice. (**A**,**C**) Representative image of hematoxylin and eosin (H/E)-stained lungs of LPS- and hyperoxia-induced lung injury in mice receiving either AVR-25 or AVR-48 treatment or non-treatment (*n* = 4–5 in each group). Scale bar = 100 µm. (**B**,**D**) Lung injury score in LPS- and hyperoxia-induced lung injury in mice receiving AVR-25 or AVR-48 treatment or non-treatment (*n* = 4–5 in each group, * *p* < 0.05 ** *p* < 0.01 and *** *p* < 0.001). AVR-25 or AVR-48 treated ALI mice showed decreased lung injury score as demonstrated by diminished pulmonary hemorrhage, peri-vascular exudates, thickened alveolar septa, and airspace edema as compared to ALI mice without treatment. Data are expressed as mean ± SEM. For statistical analysis, student’s unpaired *t*-test and one-way analysis of variance (ANOVA) followed by Tukey post hoc analysis were used. LPS: lipopolysaccharide; ALI: acute lung injury; PBS: phosphate buffered saline; RA: room air; HYP: hyperoxia.

Finally, we examined whether improved pulmonary vascular leakage was also associated with decreased lung injury in ALI mice. We assessed the local injury response by performing a detailed histological examination of the lungs from ALI mice with or without treatment. As shown in hematoxylin and eosin (H&E) staining of lung sections of LPS-induced ALI mice treated with AVR-25 or AVR-48, these mice showed decreased lung injury scores as demonstrated by diminished pulmonary hemorrhage, perivascular exudates, thickened alveolar septa, and airspace edema as compared to ALI mice without any treatment (Figure 6A,B). Consistent with lung injury scores of LPS-induced ALI mice treated with AVR-25 or AVR-48, we observed that treatment with these compounds also improved lung injury caused by hyperoxia exposure. The hyperoxia induced lung histology and injury scores, before and after treatment, are shown in Figure 6C,D, respectively. 

### 2.9. Effect of AVR-48 on CLP-Induced Survival and Lung Histopathology 

**Figure 7:**
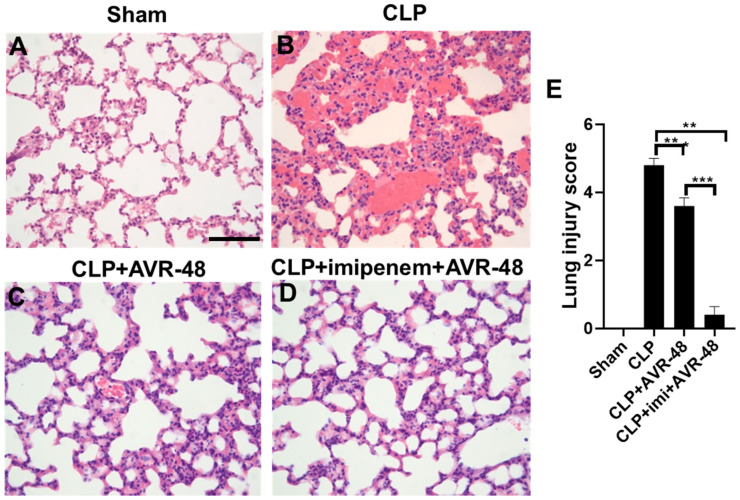
Treatment with AVR-48 showed decreased lung injury in CLP-induced ALI in mice. Representative image of H/E-stained lungs of CLP-induced lung injury in mice receiving AVR-48 treatment. (**A**) Normal lungs in the sham group. (**B**) In the CLP group, the alveolar membranes show fragmentation leading to expansion of alveolar sacs associated with severe vascular congestion, thrombosis, and hemorrhage. (**C**) Signs of lung injury are diminished after treatment with AVR-48 alone or (**D**) with a combination of imipenem + AVR-48. A CLP + imipenem group was not done here, as we published that finding earlier and observed that AVR-25 demonstrated better efficacy when given in combination with imipenem, than with imipenem alone. Scale bar = 100 µm representative of (**A**–**D**). (**E**) Right panel shows lung injury scores with severe injury in the CLP group followed by significant recovery after treatment with AVR-48 alone or with imipenem+AVR-48. ** *p* < 0.01; *** *p* < 0.001. Data are expressed as mean ± SEM. For statistical analysis, student’s unpaired *t*-test and one-way analysis of variance (ANOVA) followed by Tukey post hoc analysis were used. CLP: cecal ligation and puncture.

We have previously reported that AVR-25 (10 mg/kg, IV) mitigates the storm of inflammation and minimizes tissue injury (including lung injury) in a mouse model of CLP-induced sepsis, making it a strong potential candidate for adjunctive therapy in intra-abdominal sepsis [15]. We, therefore, wanted to test if the monosaccharide analog AVR-48, at the same dose of 10 mg/kg IV, is able to recapitulate a similar effect on lung injury following severe sepsis, in the CLP mouse model. While there was 100% mortality in the CLP group alone within 72 h of the procedure, administration of AVR-48 alone or in combination with imipenem, increased the survival to 80% (Figure S2). As CLP-induced sepsis results in multi-organ failure due to polymicrobial infection, the lungs were severely damaged in the CLP group as characterized by severe thrombosis and vascular congestion (Figure 7A,B). After treatment with AVR-48, the appearance of the lung revealed decreased inflammation and hemorrhage. The blood vessels and alveolar sacs demonstrated an appearance that was closer to that of a normal lung (Figure 7C,D). There was a significant improvement in the lung injury score in the treated groups as compared to the CLP group (*p* < 0.001) (Figure 7E). As antibiotics are the standard of care clinically, AVR-48 was tested in conjunction with imipenem. Further, as CLP-induced sepsis also leads to multi-organ failure, we analyzed the histopathological changes in the other vital organs and scored them based on their injury and recovery. As expected, all other organs showed a dramatic improvement after treatment with AVR-48 as compared to the CLP-only group. Additionally, there was a significant improvement in the lung injury score (Figure 7E) as well as injury scores in other organs (Figure S3) in the CLP + imipenem + AVR-48 group when compared to CLP + AVR-48 group (*p* < 0.001). The injury scores of all the vital organs affected by CLP-induced sepsis and recovery after AVR-48 treatment are summarized in Table S4. 

## 3. Discussion

ALI and ARDS are characterized by overproduction of pro-inflammatory cytokines such as IL-1β, TNF-α, and IL-6 as a result of bacterial or viral infection, trauma, excess exposure to oxygen and noxious gases. These mediators induce endothelial and epithelial injury in the lung, vascular leakage, and edema, subsequently causing the development of ALI and ARDS [21]. The recent COVID-19 pandemic is also associated with respiratory failure from ARDS and is the leading cause of mortality, accompanied by hyperinflammation, increased level of IL-1β and IL-6 in these patients [22,23]. Re-analysis of data from a phase 3 randomized controlled trial of IL-1 antagonist (Anakinra) in sepsis demonstrated significant survival benefit in patients with hyperinflammation, without increased adverse events [24]. Mounting evidence suggests that reducing inflammation via activation of the anti-inflammatory cytokine IL-10 could be therapeutic for ARDS. IL-10, primarily produced by T-helper 2 cells, B cells, monocytes, macrophages, and keratinocytes is known to reduce the synthesis of pro-inflammatory cytokines and terminate inflammatory responses [25]. Low levels of IL-10 are found in patients with transfusion-related ALI [26]. Importantly, treatment with IL-10 alleviated lung injury induced by ischemia-reperfusion, LPS [18], bleomycin [27], and ozone [28] and the absence of endogenous IL-10 enhanced ALI induced by carrageenan [29]. In addition to this, it was also reported that pre-incubation of cultured fetal rat alveolar type II cells with recombinant IL-10 prior to 65% hyperoxia exposure decreased cellular necrosis and increased cell proliferation [30]. As reported by us [31] and others [32], exogenous IL-10 treatment alleviated hyperoxia-induced ALI in mice, possibly by regulating neutrophil recruitment with subsequent generation of cytokines, nitric oxide (NO), and matrix metalloproteinases. IL-10 is also known to negatively regulate the production of IL-1β [33]. Here, we have demonstrated that compounds AVR-25 and AVR-48 decreased the hyperinflammation effect by not only up-regulating the compensatory anti-inflammatory cytokine IL-10, but also by decreasing the inflammatory cytokines (IL-1β, IL-6) in lung tissues and immune cells of the BALF.

ARDS is characterized by acute inflammation with subsequent neutrophil recruitment into the lung, microvascular damage, increased pulmonary vascular, and epithelial permeability causing acute respiratory failure and death [2,3,4]. Both AVR-25 and AVR-48 treatment attenuated expression of lung adhesion molecules and decreased the Evans blue dye leakage indicating the improvement in restoring permeability in the LPS- and hyperoxia- injured mouse lungs. We noted that, in the same treatment groups, there was an increase in junctional adherence proteins VE-cadherin, β-catenin, and Src that could contribute to the maintenance of healthy tight junctions in the lung epithelial cells, and not allow excess BAL protein leakage to the alveoli compromising the gas exchange, as observed in ARDS lungs. We also noted that AVR-25 and AVR-48 decrease lung cell death mediated by LPS or hyperoxia-exposure to mice. We anticipate that, with decreased inflammation, deceased lung permeability, improved pulmonary barrier function, and decreased lung cell death, AVR-25 and AVR-48 may also improve lung function and gas exchange. 

Additionally, we tested AVR-48 in the CLP-induced polymicrobial infection induced sepsis model in adult mice, which also causes ALI leading to severe ARDS. This model has been previously used by us to report that our initial chitohexaose analog, AVR-25, was very effective in increasing survival and decreasing lung and other organ injuries caused by this acute systemic infection and inflammatory condition [15]. The goal here was to determine if treatment with AVR-48, a shorter sugar chain analog with an easier large scale manufacturing synthetic route (AVR-48 has 1 monosaccharide unit compared to AVR-25 which has 6 monosaccharide units), could also minimize lung injury after CLP-induced sepsis. The decrease in lung injury score in the CLP + AVR-48 treated group was comparable to our previously reported results for CLP + imipenem, as well as CLP + AVR-25 treated groups [15]. Additionally, the significant decrease in the CLP + AVR-48 + imipenem lung injury scores demonstrated an apparent synergistic effect, as was reported for CLP + AVR-25 + imipenem treatment [15]. AVR-48 treatment also appears to contribute to improved lung function and uniform oxygen supply to other organs resulting in decreased tissue injury, as shown by the histopathological examination and scoring of all the vital organs in the CLP model, which demonstrated improvement in recovery of all key organ systems after treatment with AVR-48 in CLP mice. In several cases, sepsis survivors develop ARDS more frequently and with high severity [34]. Based on our results, we propose that the significant increase in survival of the mice (80%) in the treated CLP group could be the result of improved lung function and decreased lung and other organ tissue injury, and that AVR-48 can be used as a potential therapeutic candidate for all ALI/ARDS.

In conclusion, our data from these studies demonstrate that the LPS-, hyperoxia- and CLP-induced injury in ALI is markedly attenuated after treatment with either AVR-25 or AVR-48. Specifically, with treatment, there was decreased lung inflammation, improved pulmonary endothelial barrier function, and decreased lung injury. Both AVR-25 and AVR-48 were equally effective in combating ALI/ARDS. Due to the less complex structure for synthesis and manufacturing of AVR-48, we selected AVR-48 for further preclinical toxicology and pharmacokinetic assessment in rats (rats being considered larger models as compared to mice). AVR-48 demonstrated a good safety profile and provided an approximate 8-fold therapeutic window over the efficacious dose. Our PK study shows that the drug exerts its therapeutic effect by systemic distribution based on its availability of concentration in the plasma, lung tissue and BALF. Though we have seen similar therapeutic efficacy for both AVR-25 and AVR-48 in LPS and hyperoxia induced ALI/ARDS models, we anticipate that compound AVR-48 has the potential to be developed commercially as an important anti-inflammatory therapeutic option to improve the mortality and morbidity of ALI/ARDS. 

## 4. Materials and Methods

### 4.1. Animals

Male C57BL6/J mice (12–15 weeks old) were purchased from the Jackson Laboratory (Bar Harbor, ME, USA) and used for ALI and CLP studies. The animals were received and acclimated for two weeks before initiating the experimental studies. All mice studies were approved by the Institutional Animal Care and Use Committee (IACUC) of Drexel University College of Medicine, Philadelphia, PA. The Drexel IACUC number for the mouse study was 20705, approved on 25 July 2018. Both male and female Sprague Dawley Crl:CD (SD) rats (8–10 weeks old; Charles River Laboratories, Raleigh, NC, USA) were used for the toxicology and PK studies to evaluate safety, determine MTD and the PK profile of AVR-48. A minimum 6-day acclimation period was allowed between receipt of the animals and the start of treatment to accustom the rats to the laboratory environment. All rat studies were approved by the IACUC of ITR Laboratories, Canada. The ITR laboratory IACUC number for the rat study was 74775, approved on 20 September 2019 The sex, number of mice, and experimental condition are summarized in Table S3.

### 4.2. Chemicals and Reagents

The synthesis and structural characterization of compounds AVR-25 and AVR-48 were performed in the AyuVis Research Laboratory following in-house procedures (14). Imipenem (Primaxin^®^) was purchased from Carbosynth Inc. (San Diego, CA, USA); LPS (*Escherichia coli* O111: B4) was purchased from List Biological Labs, Inc (Campbell, CA, USA); endotoxin-free phosphate-buffered saline (PBS) was purchased from Sigma-Aldrich Inc., St. Louis, MO, USA.

### 4.3. Formulations

#### 4.3.1. Formulation for Mouse Efficacy Studies

For the mouse efficacy studies, both AVR-25 and AVR-48 were reconstituted in 0.9% sterile normal saline to give a final concentration of 10 mg/kg; 125 µL was administered intraperitoneally (IP) for hyperoxia- and LPS- induced lung injury, and IV via the lateral tail vein for CLP-induced lung injury. Imipenem (Primaxin^®^) was formulated in sterile 0.9% saline solution and administered subcutaneously (SC) at a dose of 5 mg/kg to mice in a 0.2 mL volume near the dorsal scapular region for CLP-induced lung injury study. LPS was dissolved in sterile 0.9% saline solution and 100 µL was administered intratracheally for the ALI study.

#### 4.3.2. Formulation for Rat PK/Tox Studies

For the PK study of AVR-48 in rats, a formulation consisting of 10% DMSO, 20% tetraglycol, 20% PEG 400, and 50% sterile water was developed. Each preparation was made on the same day of dose administration. The components of the control/vehicle item formulation were added sequentially. Briefly, the appropriate volume of DMSO (10% of the final volume) was measured into a container followed by gradual addition of PEG 400 (20% of the final volume) and sterile water (50% of the final volume) to make up the volume and mixed thoroughly till completely dissolved. The final solution was filtered through a 0.22 µm membrane filter (Millipore Millex^®^ GP PES, St. Louis, MO, USA) into a sterile vial.

### 4.4. Toxicology Study 

The toxicology study of AVR-48 was performed with both 8–10 week old male and female Sprague Dawley rats, in two phases following standard FDA guidance [M3(R2)R]. AVR-48 was injected IV at a volume of 5 mL/kg of body weight after reconstituting in 10% DMSO, 20% tetraglycol, and 20% PEG 400 in sterile water. In the dose escalation phase, 75 to 100 mg/kg per day was administered as two separate doses approximately 6–7 h apart by IV slow bolus injection at a volume of 5 mL/kg of body weight. Three animals of each sex were dosed at each dose level. During the repeat dose phase, daily doses of 0, 40, and 80 mg/kg/day were administered as two divided doses delivered by slow bolus injection at a volume of 5 mL/kg of body weight approximately 6–7 h apart. Study animals (*n* = 5/sex/dose) were treated for three days with a total of six doses for AVR-48. Twelve to eighteen hours after the last dose was administered, animals were euthanized and blood collected for analysis of clinical chemistry and other hematological parameters. Detailed necropsy examinations were performed on each animal, and tissues were collected and fixed with standard fixatives for histological analysis and interpretation.

### 4.5. Pharmacokinetic (PK) Study 

All PK studies were also conducted with 8–10-week-old male and female Sprague Dawley rats. An additional nine animals/sex/dose were used for PK analysis and blood (approximately 0.3 mL each) was collected from each rat on days 1 and 3 of the treatment period at time points of 5, 30, 60, 90, 120, and 180 min, and 6, 12 h, and 24 h post dose that were randomized to provide a total of three samples/sex for each time point. Samples were collected by jugular venipuncture and the samples were collected into tubes containing the anticoagulant, K_3_EDTA. The collection of BALF was limited to the terminal time points of 6 h, 12 h, and 24 h. AVR-48 concentrations in the blood and BALF were determined using LC/MS/MS analysis developed in house. PK parameters were estimated using Phoenix pharmacokinetic software (Certara, Princeton, NJ, USA) using a non-compartmental approach consistent with the IV bolus injection. 

### 4.6. Murine Model of Acute Lung Injury (ALI)

#### 4.6.1. Experimental Protocol

Three models of ALI were used in the current study: hyperoxia-, LPS-, and CLP-induced lung injury: LPS-induced ALI was induced by administering a one-time intratracheal instillation of LPS (100 µg in a volume of 100 µL). Animals were sacrificed after 24 h following LPS administration while two doses of AVR-25 and AVR-48 were administered (10 mg/kg, IP) at 4 h and 12 h following LPS dosing. Hyperoxia-induced ALI was initiated by keeping the mice in cages in airtight polypropylene chamber exposed to hyperoxia (100% oxygen) for 48 h, following a standard protocol [35]. Two doses of AVR-25 and AVR-48 were administered (10 mg/kg, IP) at 4 h and 12 h following hyperoxia exposure, and mice were sacrificed after 48 h.

For CLP-induced lung injury, CLP was performed as previously described by us [15]. As antibiotics are the standard of care in sepsis patients, one dose of imipenem was given SC at 5 mg/kg 30 min after the procedure, followed by two doses of AVR-48 (10 mg/kg, IV) at 16 h and 24 h following the surgery, and sacrificed after 72 h. The sham mice (controls) had the abdominal cavity opened, the cecum was not perforated, and the peritoneum was stitched back.

At the end of the experimental time points for all the above groups, the mice were euthanized with an overdose of a cocktail of xylazine (100 mg/kg)/ketamine (10 mg/kg, IP) and BALF and lung tissues were harvested for further analysis. Male mice were used for all the ALI experiments. Table S3 shows the summary of all animals used in different experiments in this study.

#### 4.6.2. Bronchoalveolar Lavage Fluid (BALF) Analysis 

BALF was obtained from the anesthetized mice by cannulating the upper part of the trachea with a blunt 22-gauge needle and then by lavaging three times with 1.0 mL of cold PBS (pH 7.4) [36]. The fluid recovery rate was about 90%. Lavaged samples were kept on ice, and the BALF was centrifuged at 4 °C. Total cell counts in BALF were determined using the TC20 automated cell counter (Bio-Rad Laboratories, Inc., Hercules, CA, USA). The differential cell counts were performed on cytospin preparations after staining with the Hema 3 differential Staining kit (Fisher Scientific, Kalamazoo, MI, USA). Total protein concentration in the BALF was measured using the Pierce^TM^ BCA assay kit (Fisher Scientific Co, Houston, TX, USA), as previously described [36]. 

#### 4.6.3. Assessment of Lung Endothelial Cell Permeability

Lung endothelium permeability was determined by measuring the accumulation of Evans blue in the lungs, as previously described [36]. In brief, Evans blue dye (EBD) solution (Sigma-Aldrich, Inc., St. Louis, MO, USA, 5 mg/mL) was injected into the tail vein at a concentration of 50 mg/kg before 30 min of the experimental time point. At the experimental time points, lungs were excised and weighed. EBD was extracted in 2 mL formamide kept in a water bath at 60 °C for 3 h, and the samples were centrifuged at 500 g for 10 min. The absorption of light at 595 nm was measured spectrophotometrically (Microplate reader, Bio-Rad Laboratories, Hercules, CA, USA) with the supernatants.

#### 4.6.4. Enzyme-Linked Immunosorbent Assay (ELISA)

Interleukin (IL)-6, IL-1β, and IL-10 were quantified on BALF while E-selectin was quantified in lung homogenate using commercially available DuoSet® ELISA kits (R&D Systems, Minneapolis, MN, USA) according to the manufacturer’s instructions, as previously described [36]. Briefly, Nalgene Nunc Maxisorp™ plates were coated overnight with antibodies to IL-6 (2 µg/mL), IL-1β (2 µg/mL) IL-10 (2 µg/mL), or E-selectin (2 µg/mL) and the following morning plates were washed and blocked for 2 h. Samples were added to the wells at various dilutions and then incubated with detection antibody for 2 h. Plates were subsequently washed, and streptavidin-HRP conjugate antibody was added to each well for 20 min. This was followed by an additional wash step and finally plates were incubated with a substrate solution and the enzymatic reaction was then quantified by measuring absorbance at 450 nm using a standard plate reader (Biotek Instrument, Inc., Winooski, VT, USA).

### 4.7. Western Blotting

Western blotting was done following the methodology as described previously [36]. In brief, lung tissues were homogenized, and proteins were extracted using ice-cold RIPA buffer containing protease inhibitors (Roche cOmplete™ Mini) and phosphatase inhibitors (Roche cOmplete Mini). Lung homogenate were centrifuged (14,000× *g*) at 4 °C for 15 min and the supernatant was collected for further analysis. Thirty micrograms of protein were loaded onto each well, separated on 10% SDS-polyacrylamide gel and then transferred onto a nitrocellulose membrane (Bio-Rad, Hercules, CA, USA) using a Bio-Rad Mini-Blot transfer apparatus. Immunoblotting was performed at 4 °C overnight using primary antibodies directed against IL-6 (Dilution 1/200, Santa Cruz Biotechnology, Inc., Dallas, TX, USA), IL-1β (Dilution 1/1000, Cell Signaling Technology, Danvers, MA, USA), ICAM-1 (dilution 1/1000, R&D Systems, Minneapolis, MN, USA), VCAM-1 (dilution 1/1000, Cell Signaling Technology, Danvers, MA, USA), E-selectin (dilution 1/1000, Abcam, Cambridge, MA, USA), VE-cadherin (dilution 1/500, Cell Signaling Technology, Danvers, MA, USA), β-Catenin (dilution 1/1000, Cell Signaling Technology, Danvers, MA, USA), p-Src (dilution 1/1000, Cell Signaling Technology, Danvers, MA, USA), Src (dilution 1/1000, Cell Signaling Technology, Danvers, MA, USA), cleaved caspase-3 (dilution 1/500, Cell Signaling Technology, Danvers, MA, USA), gapdh (dilution 1/1000, Cell Signaling Technology, Danvers, MA, USA), Vinculin (dilution 1/1000, Cell Signaling Technology, Danvers, MA, USA). Membranes were then incubated with a 1:10,000 dilution of secondary antibodies (IRDye 800CW Goat anti-Mouse IgG (P/N 926-32210 and IRDye 800CW Goat anti-Rabbit IgG (P/N 926-32211, Li-Cor Biosciences, Lincoln, NE, USA) at room temperature for 1h. Membranes were washed with Tris-PBS three times and finally with PBS. Immunoblots were visualized using the Odyssey infrared imaging system (Li-Cor Biosciences, Lincoln, NE, USA). The band intensity on the western blots were analyzed and quantified using the Image J software (Version 1.6, National Institutes of Health, MD, USA). 

### 4.8. Terminal Deoxynucleotidy1 Transferase-Mediated dUTP Nick End-Labeling (TUNEL) Assay

Apoptosis in lungs tissues were confirmed by TUNEL assay using the fluorescence based TUNEL Kit (In Situ Cell Death Detection Kit, Roche Diagnostics, Indianapolis, IN, USA) according to the manufacturer’s instructions. In brief, de-paraffinized lung tissue sections were permeabilized using 0.3% Triton-X 100, blocked with 1% BSA and then incubated with TUNEL reaction mixture (1 part of enzyme solution and 9 parts of label solution) for 1h at 37 °C in the dark. Slides were washed with PBS, mounted with VECTASHIELD® mounting medium with DAPI (Catalog no. H-1800-10, Vector Laboratories, Burlingame, CA, USA) and visualized with a fluorescent microscope using an exciting wavelength of 460–490 nm. Percentage TUNEL positive cells were expressed as total number of green positive cells/total number of DAPI positive cells. 

### 4.9. Histological Analysis

H&E staining was performed on 5 µm paraffin lung tissue sections following standardized methods. Four to five random images per lung and 4–5 lungs per experimental group were characterized for lung injury score analysis.

### 4.10. Lung Injury Scoring 

Two different scoring systems were used for lung injury scoring, as the experiments were done at different times. The LPS-/hyperoxia-induced ALI scoring was done by DS, while the CLP-induced lung and other organs’ scoring was done by two additional independent investigators (PD and BA). For the hyperoxia- and LPS-induced lung injury, scoring was done on histological sections based on neutrophil infiltration and epithelial injury using the methods as described before with modifications [37,38]. Four to five random images per lung and 4–5 lungs per experimental group were characterized for lung injury score analysis. We used a 0–4 scoring system in which 0 = no infiltration/injury, 1 = minimal infiltration/injury to 25% of the field, 2 = mild infiltration/injury 12 to 50% of the field, 3 = moderate infiltration/injury to 75% of the field, and 4 = severe infiltration/diffuse injury. For the CLP-induced ALI, scoring was done following the methodology as reported earlier [39] by a qualified pathologist (BA) in masked manner. Lungs were harvested from all the mice after the endpoint, and lung injury was scored based on the criteria of alveolar damage and vascular congestion and hemorrhage. As CLP-induced sepsis also results in damage to the rest of the viscera, which can contribute to ALI/ARDS, other organs were also scored as above, to determine the recovery potential of AVR-48 as a candidate drug. The following (cells) were taken into account while scoring the injury level followed by recovery after treatment in other vital organs: heart (myocardium), kidney (glomeruli, tubules), gut (mucosal cells, enterocytes), spleen (lymphoid follicles), liver (hepatocytes), brain (neuronal damage), testis (Seminiferous tubules), and lymph nodes. The final scores were calculated on the basis of total percentage of the damaged/recovered area and by the number of areas/High power field (HPF) as reported above [39].

### 4.11. Statistical Analysis

Statistics were performed using GraphPad Prism 8.0 software. Two-group comparisons were analyzed by unpaired Student’s *t*-test and multiple-group comparisons were performed using one-way analysis of variance (ANOVA) followed by Tukey post hoc analysis. Survival was analyzed using Kaplan Meier plots. For the toxicology and PK study, a generalized Analysis of Covariance/Variance (ANOVA/ANCOVA) test was performed on the numerical data (three or more animals/groups) on the study as follows: An automatic transformation was used to analyze the data for homogeneity of variance using Levene’s test. Parametric and non-parametric trends were analyzed using the Williams and the Shirley–Williams tests, respectively. Homogeneous data was analyzed using the ANOVA/ANCOVA, and the significance of intergroup differences between the control and test item-treated groups was analyzed using Dunnett’s test. Heterogeneous data was analyzed using Kruskal–Wallis test and the significance of intergroup differences between the control and test item-treated groups was assessed using a nonparametric Dunnett’s test. All data are reported as mean ± SEM. A significance level of *p* < 0.05 at 95% confidence intervals was considered statistically significant for all the experiments reported in this study. 

## Data Availability

The raw data presented in this study are available in www.mdpi.com/xxx/s1.

## Supplementary Materials

The following are available online at https://www.mdpi.com/1422-0067/22/5/2573/s1, Supplemental Methods: Survival. Figure S1: PK profile of AVR-48 in Sprague Dawley rats. Figure S2: AVR-48 increases survival in adult mice. Figure S3: Histopathology of the other vital organs after AVR-48 treatment. Table S1: Individual AVR-48 toxicokinetic parameters in Sprague-Dawley rat plasma after 40 mg/Kg, 80 mg/kg IV injection of AVR-48 on Day 1. Table S2: Individual AVR-48 toxicokinetic parameters in Sprague-Dawley Rat plasma after 40 mg/kg, 80 mg/kg IV injection of AVR-48 on Day 3. Table S3: Summary of animals used for different experimental groups. Table S4: Injury Scores of different organs after CLP (represented by Mean ± SEM). Raw data.

## References

[1] Salim A., Martin M., Constantinou C., Sangthong B., Brown C., Kasotakis G., Demetriades D., Belzberg H. (2006). Acute respiratory distress syndrome in the trauma intensive care unit: Morbid but not mortal. Arch Surg..

[2] Matthay M.A., Ware L.B., Zimmerman G.A. (2012). The acute respiratory distress syndrome. J. Clin. Investig..

[3] Rubenfeld G.D., Caldwell E., Peabody E., Weaver J., Martin D.P., Neff M., Stern E.J., Hudson L.D. (2005). Incidence and outcomes of acute lung injury. N. Engl. J. Med..

[4] Mora-Rillo M., Arsuaga M., Ramirez-Olivencia G., de la Calle F., Borobia A.M., Sanchez-Seco P., Lago M., Figueira J.C., Fernandez-Puntero B., Viejo A. (2015). Acute respiratory distress syndrome after convalescent plasma use: Treatment of a patient with Ebola virus disease contracted in Madrid, Spain. Lancet Respir. Med..

[5] Villar J., Blanco J., Kacmarek R.M. (2016). Current incidence and outcome of the acute respiratory distress syndrome. Curr. Opin. Crit. Care.

[6] Matthay M.A., McAuley D.F., Ware L.B. (2017). Clinical trials in acute respiratory distress syndrome: Challenges and opportunities. Lancet Respir. Med..

[7] Zhang Z., Chen L., Ni H. (2015). The effectiveness of Corticosteroids on mortality in patients with acute respiratory distress syndrome or acute lung injury: A secondary analysis. Sci. Rep..

[8] Bernard G.R., Wheeler A.P., Russell J.A., Schein R., Summer W.R., Steinberg K.P., Fulkerson W.J., Wright P.E., Christman B.W., Dupont W.D. (1997). The effects of ibuprofen on the physiology and survival of patients with sepsis. The Ibuprofen in Sepsis Study Group. N. Engl. J. Med..

[9] Chen M., Lu J., Chen Q., Cheng L., Geng Y., Jiang H., Wang X. (2017). Statin in the treatment of ALI/ARDS: A systematic review and Meta-analysis based on international databases. Zhonghua Wei Zhong Bing Ji Jiu Yi Xue.

[10] Afshari A., Brok J., Moller A.M., Wetterslev J. (2010). Inhaled nitric oxide for acute respiratory distress syndrome (ARDS) and acute lung injury in children and adults. Cochrane Database Syst. Rev..

[11] Panda S.K., Kumar S., Tupperwar N.C., Vaidya T., George A., Rath S., Bal V., Ravindran B. (2012). Chitohexaose activates macrophages by alternate pathway through TLR4 and blocks endotoxemia. PLoS Pathog..

[12] Martinez F.O., Helming L., Gordon S. (2009). Alternative activation of macrophages: An immunologic functional perspective. Annu. Rev. Immunol..

[13] Mosser D.M., Edwards J.P. (2008). Exploring the full spectrum of macrophage activation. Nat. Rev. Immunol..

[14] Acharya S., Das P., Agarwal B. (2020). Novel Immunodulating Small Molecules. US Patent.

[15] Das P., Panda S.K., Agarwal B., Behera S., Ali S.M., Pulse M.E., Solomkin J.S., Opal S.M., Bhandari V., Acharya S. (2019). Novel Chitohexaose Analog Protects Young and Aged mice from CLP Induced Polymicrobial Sepsis. Sci. Rep..

[16] Iscimen R., Cartin-Ceba R., Yilmaz M., Khan H., Hubmayr R.D., Afessa B., Gajic O. (2008). Risk factors for the development of acute lung injury in patients with septic shock: An observational cohort study. Crit. Care Med..

[17] Armstrong L., Millar A.B. (1997). Relative production of tumour necrosis factor alpha and interleukin 10 in adult respiratory distress syndrome. Thorax.

[18] Bi M.H., Wang B.E., Zheng X.X., Li M., Mayer K., Zhang S.W. (2008). The effect of recombinant interleukin-10/Fc fusion protein on lipopolysaccharide-induced acute lung injury in mice. Zhongguo Wei Zhong Bing Ji Jiu Yi Xue.

[19] Inoue G. (2000). Effect of interleukin-10 (IL-10) on experimental LPS-induced acute lung injury. J. Infect. Chemother..

[20] Hiroshima Y., Hsu K., Tedla N., Chung Y.M., Chow S., Herbert C., Geczy C.L. (2014). S100A8 induces IL-10 and protects against acute lung injury. J. Immunol..

[21] Densmore J.C., Signorino P.R., Ou J., Hatoum O.A., Rowe J.J., Shi Y., Kaul S., Jones D.W., Sabina R.E., Pritchard K.A. (2006). Endothelium-derived microparticles induce endothelial dysfunction and acute lung injury. Shock.

[22] Chien J.-Y., Hsueh P.-R., Cheng W.-C., Yu C.-J., Yang P.-C. (2006). Temporal changes in cytokine/chemokine profiles and pulmonary involvement in severe acute respiratory syndrome. Respirology.

[23] Conti P., Ronconi G., Caraffa A., Gallenga C.E., Ross R., Frydas I., Kritas S.K. (2020). Induction of pro-inflammatory cytokines (IL-1 and IL-6) and lung inflammation by Coronavirus-19 (COVI-19 or SARS-CoV-2): Anti-inflammatory strategies. J. Biol. Regul. Homeost Agents.

[24] Shakoory B., Carcillo J.A., Chatham W.W., Amdur R.L., Zhao H., Dinarello C.A., Cron R.Q., Opal S.M. (2016). Interleukin-1 Receptor Blockade Is Associated With Reduced Mortality in Sepsis Patients With Features of Macrophage Activation Syndrome: Reanalysis of a Prior Phase III Trial. Crit. Care Med..

[25] Moore K.W., Rousset F., Banchereau J. (1991). Evolving principles in immunopathology: Interleukin 10 and its relationship to Epstein-Barr virus protein BCRF1. Springer Semin. Immunopathol..

[26] Kapur R., Kim M., Rebetz J., Rondina M.T., Porcelijn L., Semple J.W. (2017). Low levels of interleukin-10 in patients with transfusion-related acute lung injury. Ann. Transl. Med..

[27] Arai T., Abe K., Matsuoka H., Yoshida M., Mori M., Goya S., Kida H., Nishino K., Osaki T., Tachibana I. (2000). Introduction of the interleukin-10 gene into mice inhibited bleomycin-induced lung injury in vivo. Am. J. Physiol. Lung Cell Mol. Physiol..

[28] Reinhart P.G., Gupta S.K., Bhalla D.K. (1999). Attenuation of ozone-induced lung injury by interleukin-10. Toxicol. Lett..

[29] Cuzzocrea S., Mazzon E., Dugo L., Serraino I., Di Paola R., Genovese T., De Sarro A., Caputi A.P. (2002). Absence of endogenous interleukin-10 enhances the evolution of acute lung injury. Eur. Cytokine Netw..

[30] Lee H.-S., Kim C.-K. (2011). Effect of recombinant IL-10 on cultured fetal rat alveolar type II cells exposed to 65%-hyperoxia. Respir. Res..

[31] Bhandari V. (2008). Molecular mechanisms of hyperoxia-induced acute lung injury. Front Biosci..

[32] Li H.D., Zhang Q.X., Mao Z., Xu X.J., Li N.Y., Zhang H. (2015). Exogenous interleukin-10 attenuates hyperoxia-induced acute lung injury in mice. Exp. Physiol..

[33] Sun Y., Ma J., Li D., Li P., Zhou X., Li Y., He Z., Qin L., Liang L., Luo X. (2019). Interleukin-10 inhibits interleukin-1β production and inflammasome activation of microglia in epileptic seizures. J. Neuroinflammation.

[34] Sheu C.-C., Gong M.N., Zhai R., Chen F., Bajwa E.K., Clardy P.F., Gallagher D.C., Thompson B.T., Christiani D.C. (2010). Clinical Characteristics and Outcomes of Sepsis-Related vs Non-Sepsis-Related ARDS. CHEST.

[35] Syed M., Das P., Pawar A., Aghai Z.H., Kaskinen A., Zhuang Z.W., Ambalavanan N., Pryhuber G., Andersson S., Bhandari V. (2017). Hyperoxia causes miR-34a-mediated injury via angiopoietin-1 in neonatal lungs. Nat. Commun..

[36] Shah D., Romero F., Stafstrom W., Duong M., Summer R. (2013). Extracellular ATP mediates the late phase of neutrophil recruitment to the lung in murine models of acute lung injury. Am. J. Physiol. Lung. Cell Mol. Physiol..

[37] Matute-Bello G., Downey G., Moore B.B., Groshong S.D., Matthay M.A., Slutsky A.S., Kuebler W.M., Acute Lung Injury in Animals Study G. (2011). An official American Thoracic Society workshop report: Features and measurements of experimental acute lung injury in animals. Am. J. Respir. Cell Mol. Biol..

[38] An X., Sun X., Hou Y., Yang X., Chen H., Zhang P., Wu J. (2019). Protective effect of oxytocin on LPS-induced acute lung injury in mice. Sci. Rep..

[39] Kubiak B.D., Albert S.P., Gatto L.A., Snyder K.P., Maier K.G., Vieau C.J., Roy S., Nieman G.F. (2010). Peritoneal negative pressure therapy prevents multiple organ injury in a chronic porcine sepsis and ischemia/reperfusion model. Shock.

