# Supplementary material for: Small Immunomodulatory Molecules as Potential Therapeutics in Experimental Murine Models of Acute Lung Injury (ALI)/Acute Respiratory Distress Syndrome (ARDS)
Source: Int J Mol Sci. 2021 Mar 4;22(5):2573. doi: 10.3390/ijms22052573 (PMC7961996; doi:10.3390/ijms22052573)

Small immunomodulatory molecules as potential therapeutics in experimental murine models of Acute Lung Injury (ALI)/Acute Respiratory Distress Syndrome (ARDS)

Dilip Shah[a,1,2], Pragnya Das[a,1,2], Suchismita Acharya[b,c] , Beamon Agarwal[d], Dale J Christensen[e,f], Stella M Robertson[b,g] and Vineet Bhandari[a,1]*

[a]Department of Pediatrics, Division of Neonatology, Drexel University, Philadelphia, PA, USA

[b]AyuVis Research Inc., 1120 South Freeway, Fort Worth, TX, USA

[c]Pharmacology & Neuroscience, University of North Texas Health Science Center, Fort Worth, TX, USA

[d]GenomeRxUS, Secane, PA, USA

[e]Dale J. Chistensen Consulting LLC, Cary, NC, USA

[f]Duke University Medical Center, Department of Medicine, Division of Hematology, Durham, NC
[g]Arrochar Consulting LLC, Fort Worth, TX, USA

Corresponding author*: bhandari-vineet@cooperhealth.edu

Children's Regional Hospital at Cooper, Suite# Dorrance 755, 1 Cooper Plaza, Camden, NJ 08103, Tel: 856-342-2000 extn: 1006156; Fax: 856-342-8007

[1]Current affiliation: Cooper University Health Care and Hospital, Suite 206, 401 Haddon Avenue, Education and Research Building, Camden, NJ 08103, USA

[2]These authors equally contributed to this research.

Supplemental Section

Supplemental methods

Survival

For the survival study of CLP-induced ALI, mice were injected with 2 doses of AVR-48 (IV) followed by 1 dose of imipenem (SC) as described in the CLP methodology, and allowed to survive for 10 days. Sterility was maintained throughout the procedure by operating the animals under a laminar hood. Following the procedure, the animals were returned back to the animal room for subsequent monitoring and follow up. The body temperature was recorded every 24h and feeding and social behavior was also observed. They were fed with semisolid food for 48h before resuming their regular chow diet. All mice were sacrificed after 10 days. (N=3 for sham and 5 for the rest of the groups). A CLP + imipenem group was not done in this study, as we have published this group and the findings that, AVR-25 (12) shows the best result when given in combination with imipenem, better than CLP + imipenem alone. Histopathology of the vital organs was performed and scored as previously published (37, 38) and noted in the methods section of this paper.

Supplemental Table 1: Individual AVR-48 toxicokinetic parameters in Sprague-Dawley rat plasma after 40mg/Kg, 80 mg/kg IV injection of AVR-48 on Day 1

Dose (mg/kg)	Gender	Tmax (hr)	Cmax ± SE (ng/mL)	Cmax ± SE (μM)	$AUC_{(0-t)}$ ± SE (hr*ng/mL)	$T_{1/2}$ (hr)	Cl (mL/hr/kg)	Vd (mL/kg)
40	Female	0.0833	129000 ± 3360	376.85 ± 9.81	53900 ± 2860	1.40	745	1510
40	Male	0.0833	120000 ± 3050	350.56 ± 8.91	49800 ± 962	1.64	806	1910
80	Female	0.0833	237000 ± 1700	692.37 ± 4.96	101000 ± 2350	1.45	806	1680
80	Male	0.0833	276000 ±1900	806.31 ± 5.55	118000 ± 3050	2.05	680	2020

NR: Result not reported because extrapolation exceeds 20%, or R-squared is less than 0.800;
SE: Standard Error

Supplemental Table 2: Individual AVR-48 toxicokinetic parameters in Sprague-Dawley Rat plasma after 40mg/kg, 80 mg/kg IV injection of AVR-48 on Day 3

Dose (mg/kg)	Gender	Tmax (h)	Cmax ± SE (ng/mL)	Cmax ± SE (μM)	$AUC_{(0-t)}$ ± SE (h*ng/mL)	$T_{1/2}$ (hr)	Cl (mL/h/kg)	Vd (mL/kg)	R_{AUC} (RATIO)
40	Female	0.0833	90400 ± 27900	264.09 ± 81.5	36600 ± 7040	2.34	1100	3720	0.679
40	Male	0.0833	115000 ± 12900	335.96 ± 37.68	46200 ± 3270	2.16	870	2710	0.928
80	Female	0.0833	227000 ± 18800	663.16 ± 54.92	95800 ± 5050	2.05	840	2480	0.953
80	Male	0.0833	190000 ± 40900	555.06 ± 119.48	91500 ± 9770	1.42	880	1800	0.777

R_{AUC}: Day 3 $AUC_{(0-t)}$ / Day 1 $AUC_{(0-t)}$; NR: Result not reported because extrapolation exceeds 20%, or R-squared is less than 0.800; SE: Standard Error

Supplemental Table 3: Summary of animals used for different experimental groups.

Expt No.	Experimental group	No. of animals	Drug used	Route of administration	Dose of administration	Endpoint
1	LPS-induced ALI<br>PBS-control	Mice<br>N=6<br>N=5	AVR-25	LPS-IT<br>AVR-25-IP	LPS-1 dose<br>AVR-25 (2 doses: 4h, 12h)	24h
2	LPS-induced ALI<br>PBS-control	Mice<br>N=6<br>N=5	AVR-48	LPS-IT<br>AVR-48-IP	LPS-1 dose<br>AVR-48 (2 doses: 4h, 12h)	24h
3	Hyp- induced ALI<br>RA-control	Mice<br>N=6<br>N=5	AVR-25	Mice in Hyp chamber<br>AVR-25-IP	AVR-25 (2 doses: 4h, 12h)	48h
4	Hyp- induced ALI<br>RA-control	Mice<br>N=6<br>N=5	AVR-48	Mice in Hyp chamber<br>AVR-48-IP	AVR-48 (2 doses: 4h, 12h)	48h
5	CLP-induced ALI<br>**i**.Sham control-No CLP<br>**ii.**CLP<br>**iii**.CLP+ AVR-48<br>**iv**.CLP+imipenem+ AVR-48	Mice<br>N=3<br>N=5<br>N=5<br>N=5	AVR-48	CLP procedure on mice<br><br>AVR-48, IV<br>AVR-48, IV + imipenem (SC)	AVR-48 (2 doses: 16h, 24h)<br>AVR-48 (2 doses: 16h, 24h)<br>imipenem (1 dose: 30min)	72h
6	CLP-induced Survival<br>**i**.Sham control-No CLP<br>**ii**.CLP<br>**iii**.CLP+AVR-48<br>**iv**.CLP+imipenem+AVR-48	Mice<br>N=3<br>N=5<br>N=5<br>N=5	AVR-48	AVR-48, IV<br>AVR-48, IV + imipenem (SC)	AVR-48 (2 doses: 16h, 24h)<br>AVR-48 (2 doses: 16h, 24h)<br>imipenem (1 dose: 30min)	10 days
7	Toxicity study	Rats (N=24; M+F)	AVR-48	IV	i) AVR-48 (40mg/kg, 80mg/kg)- single dose<br>ii) AVR-48 (40mg/kg, 80 mg/kg)- twice a day, for 3 days	4 days
8	Pharmacokinetic study	Rats (N=18; M+F)	AVR-48	IV	AVR-48 (40mg/kg, 80 mg/kg)- twice a day, for 3 days	***Blood drawn at*** 0.5 min, 1h, 1.5h, 2h, 3h, 6h, 12h, 24h<br><br>1 day & 3 days

Expt- Experiment; **PBS-** Phosphate buffered saline; **LPS**- Lipopolysaccharide; **IT-** intratracheal; **RA**- Room air; **Hyp-** Hyperoxia; **IP-** intraperitoneal; **IV-** intravenous; **CLP-** cecal ligation and puncture; **ALI-** acute lung injury; **M**- male; **F-** female; **N**- number; **h-** hours. **Mice**- C57BL6/J mice, male. **Rats**-Sprague-Dawley rats, male+ female.

Supplemental Table 4: Injury Scores of different organs after CLP (represented by Mean ± SEM).

Tissues		Sham	CLP	CLP+AVR-48	CLP+imipenem+AVR-48
**Heart**	Myocardial Damage	0±0	4.8±0.2	3.4±0.245	0.4±0.245
	Vascular Congestion & Hemorrhage	0±0	4.8±0.2	3.4±0.4	0.6±0.245
**Lung**	Alveolar Damage	0±0	4.8±0.2	3.6±0.245	0.2±0.2
	Vascular Congestion and Hemorrhage	0±0	4.8±0.2	3.6±0.245	0.4±0.245
**Liver**	Hepatocyte Injury	0±0	4.8±0.2	3.6±0.245	0.4±0.245
	Vascular Congestion & Hemorrhage	0±0	4.8±0.2	3.6±0.245	0.6±0.245
**Spleen**	Vascular Congestion & Hemorrhage	0±0	4.8±0.2	3.6±0.245	0.4±0.245
**Kidney**	Glomerular Injury	0±0	4.8±0.2	3.6±0.245	0.4±0.245
	Glomerular Damage	0±0	4.8±0.2	3.6±0.245	0.4±0.245
	Vascular Congestion & Hemorrhage	0±0	4.8±0.2	3.6±0.245	0.4±0.245
**Gut**	MVD	0±0	4.8±0.2	3.6±0.245	0.2±0.2
	Vascular Congestion & Hemorrhage	0±0	4.8±0.2	3.6±0.245	0.2±0.2
**Lymph Node**	Follicular Damage	0±0	4.8±0.2	3.4±0.245	0.4±0.245
**Brain**	Neuronal Damage	0±0	4.8±0.2	3.6±0.245	0.4±0.245
	Vascular Congestion & Hemorrhage	0±0	4.8±0.2	3.4±0.245	0.6±0.245
**Testis**	Tubular Damage	0±0	4.8±0.2	3.6±0.245	0.4±0.245
	Vascular Congestion & Hemorrhage	0±0	4.8±0.2	3.6±0.245	0.2±0.2

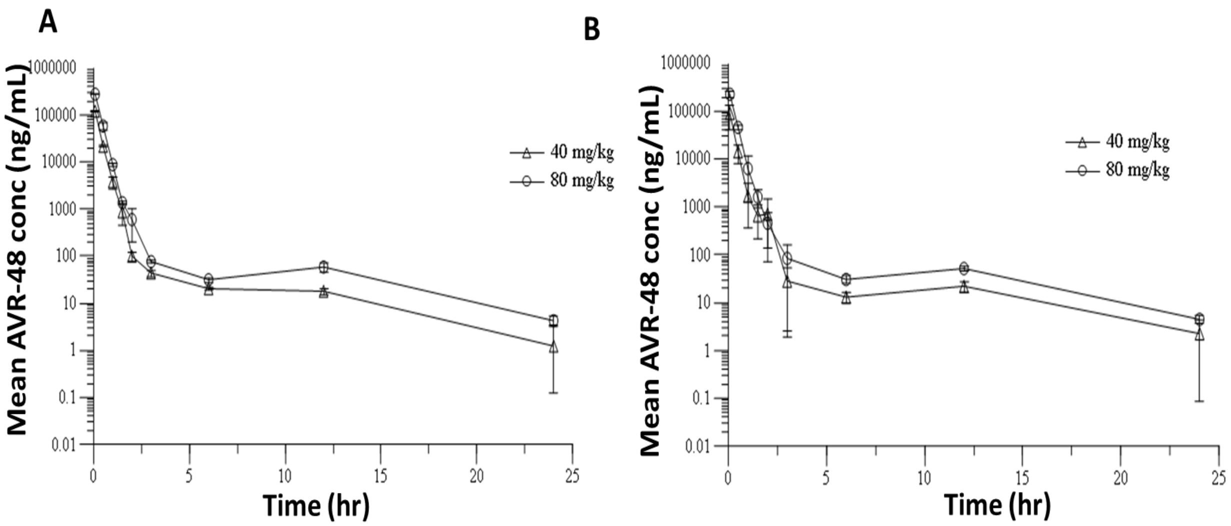

Supplemental Figure S1: PK profile of AVR-48 in Sprague Dawley rats. **A**) Concentration of AVR-48 after IV dosing of 40 mg/kg and 80mg/kg of AVR-48 on day 1 post injection at different time points. **B**) Concentration of AVR-48 after IV dosing of 40 mg/kg and 80mg/kg of AVR-48 on day 3 post injection at different time points. The data is presented as average values obtained from 3 males and 3 female rats ± SEM.

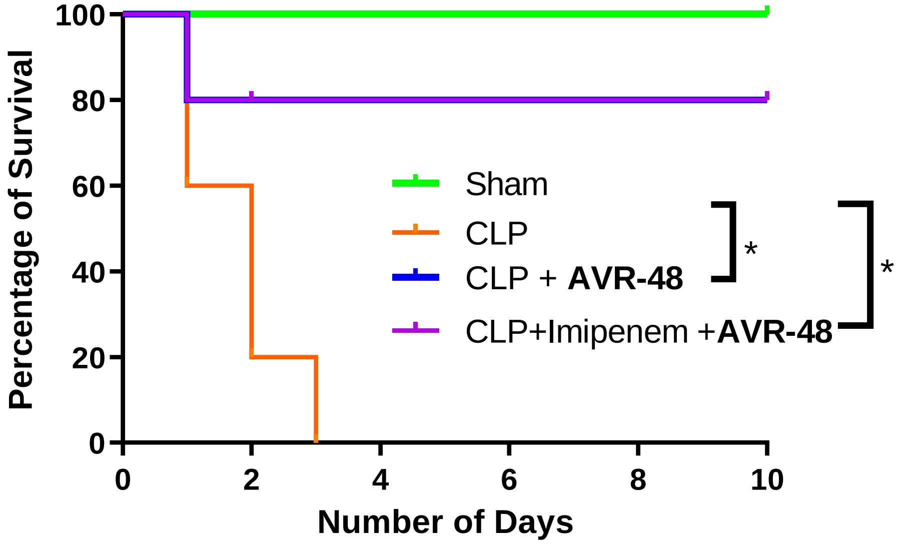

Supplemental Figure S2: AVR-48 increases survival in adult mice. Following CLP, the AVR-48 + imipenem treated group demonstrated similar survival (~80%) pattern as the group treated with AVR-48 alone. There was no mortality in the sham group, while in the CLP group there was 100% mortality within 72h of the procedure, (N = 3-5) *P<0.05.

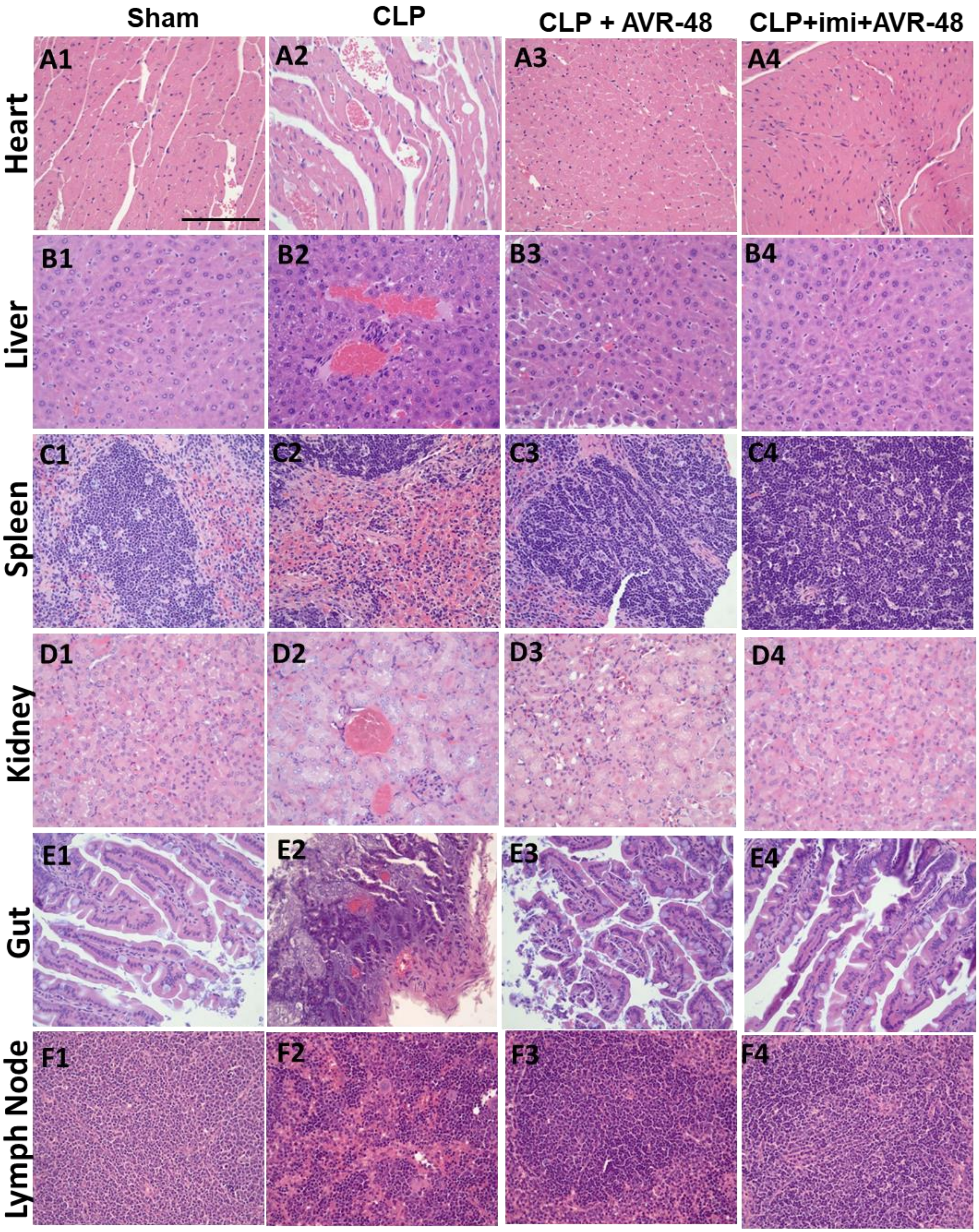
Sham
CLP
CLP + AVR-48
CLP+imi+AVR-48
Heart
Liver
Spleen
Kidney
Gut
Lymph Node
A1
A2
A3
A4
B1
B2
B3
B4
C1
C2
C3
C4
D1
D2
D3
D4
E1
E2
E3
E4
F1
F2
F3
F4

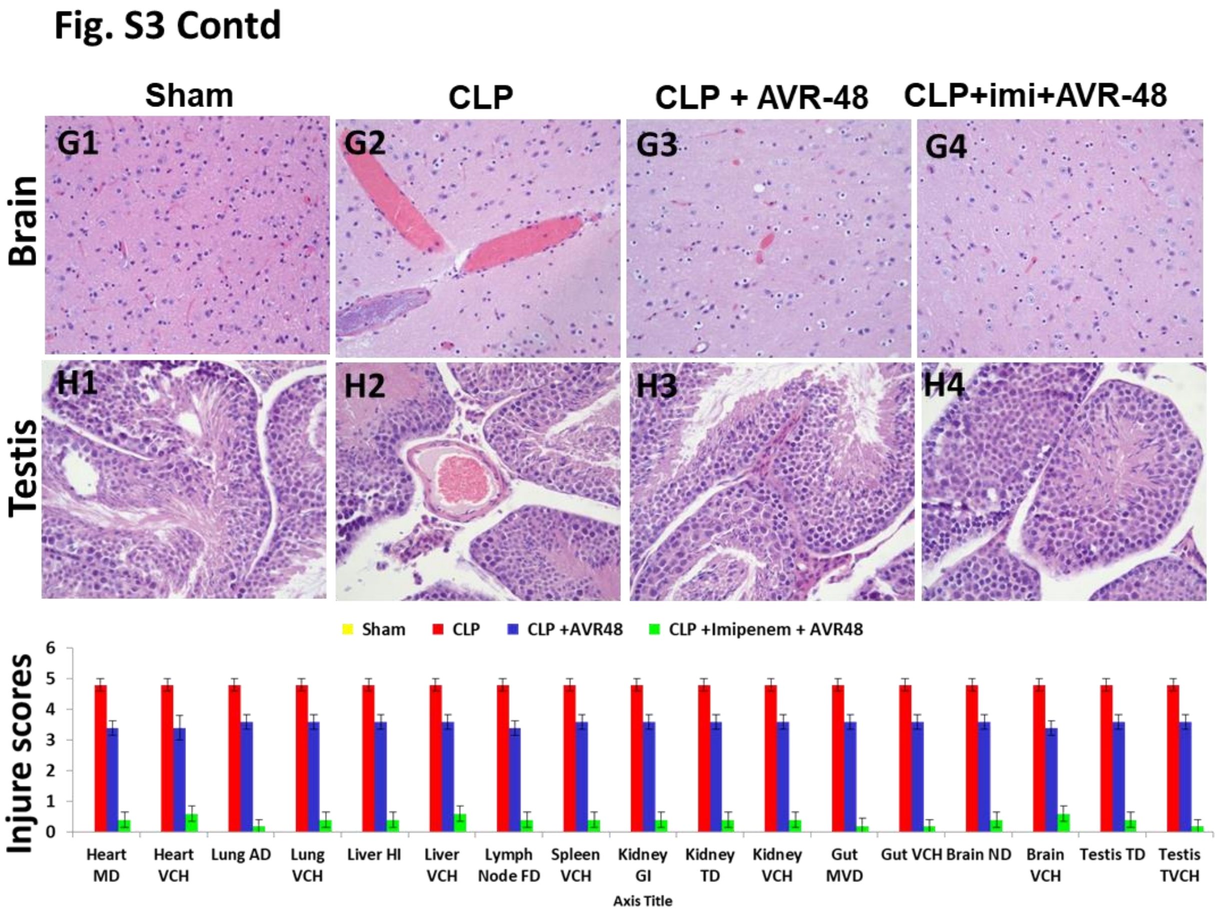

Supplemental Figure S3: Histopathology of the other vital organs after AVR-48 treatment. In the heart, the myocardium is damaged and there is vascular congestion (**A1-A2**); In the liver, hepatocytic injury is characterized by cytoplasmic damage and variation in nuclear shape and size associated with vascular congestion with hemorrhage (**B1-B2**); in the spleen, the lymphoid follicles show destruction (**C1-C2**); in the kidney, there is glomerular injury with reduction in size and damaged tubules demonstrated by lack of nuclei associated with vascular congestion with hemorrhage (**D1-D2**); in the small intestine (gut), the villi show destruction associated with vascular congestion with hemorrhage (**E1-E2**); the lymph nodes show similar injury after CLP (**F1-F2);** in the brain, there is neuronal damage, accompanied by islands of vascular congestion and hemorrhage (**G1-G2**); in the testis the seminiferous tubules are destroyed with lack of mature

spermatocytes and spermatids and associated with vascular congestion with hemorrhage (**H1-H2**). In summary, CLP induces vascular congestion and hemorrhage in all the above organs, which have a more normal appearance after treatment with AVR-48 alone (**A3-H3**), or in combination with imipenem (**A4-H4**). Scale bar =100µm and is representative of all the figures from A1-H4. Bottom panel shows injury scoring after CLP, followed by treatment with AVR-48 or imipenem+AVR-48. In all the organs, there is maximum damage after CLP which is significantly recovered after treatment with AVR-48 alone (**p<0.01) or with imipenem+AVR-48 (***p<0.001); imi=imipenem.

Uncut Immunoblots used in the main Figures

Full uncut immunoblot for IL-6 used in the main **Figure 1F**. Blue box indicates the lanes of IL-6 immunoblots used in the figure.

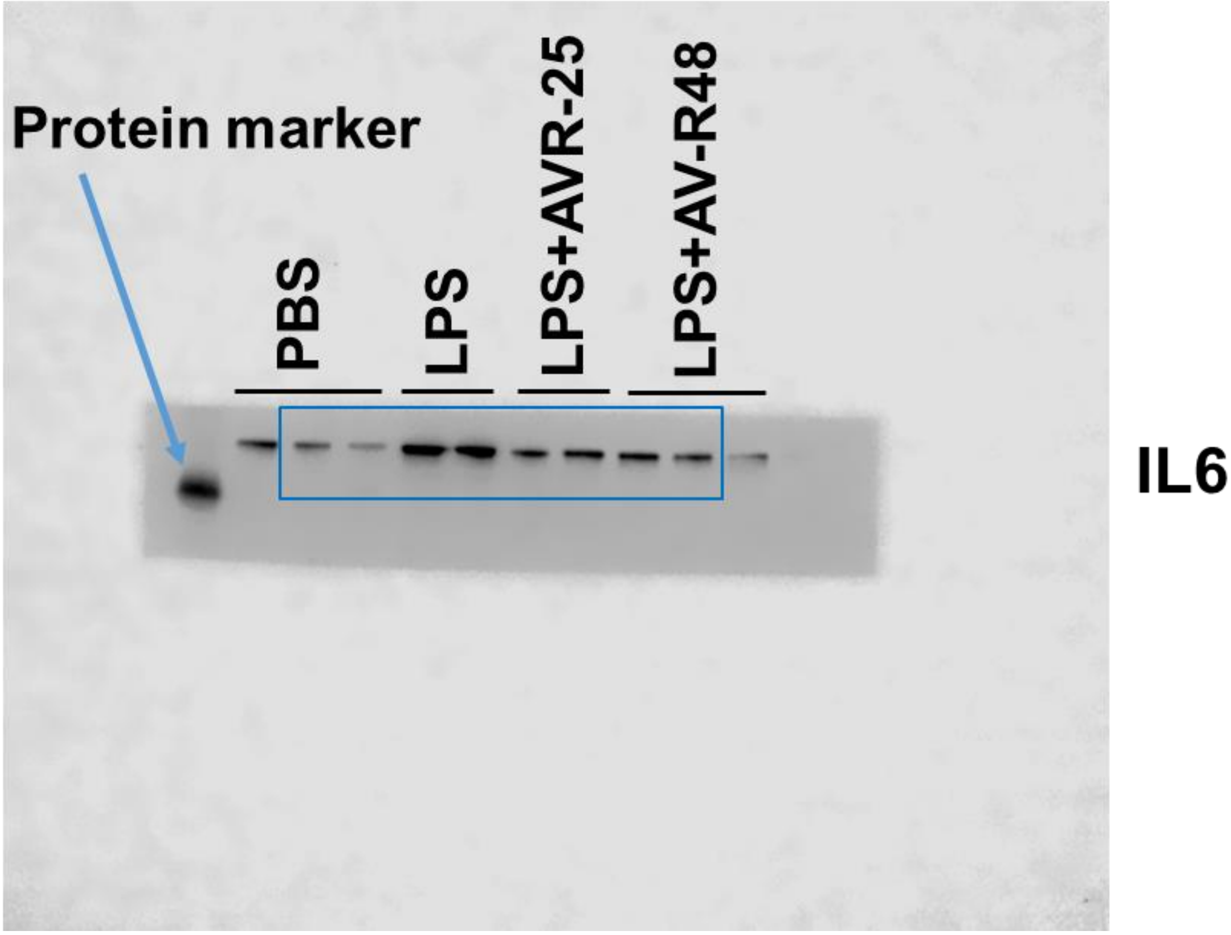

Full uncut immunoblot for IL-1β used in the main **Figure 1F**.

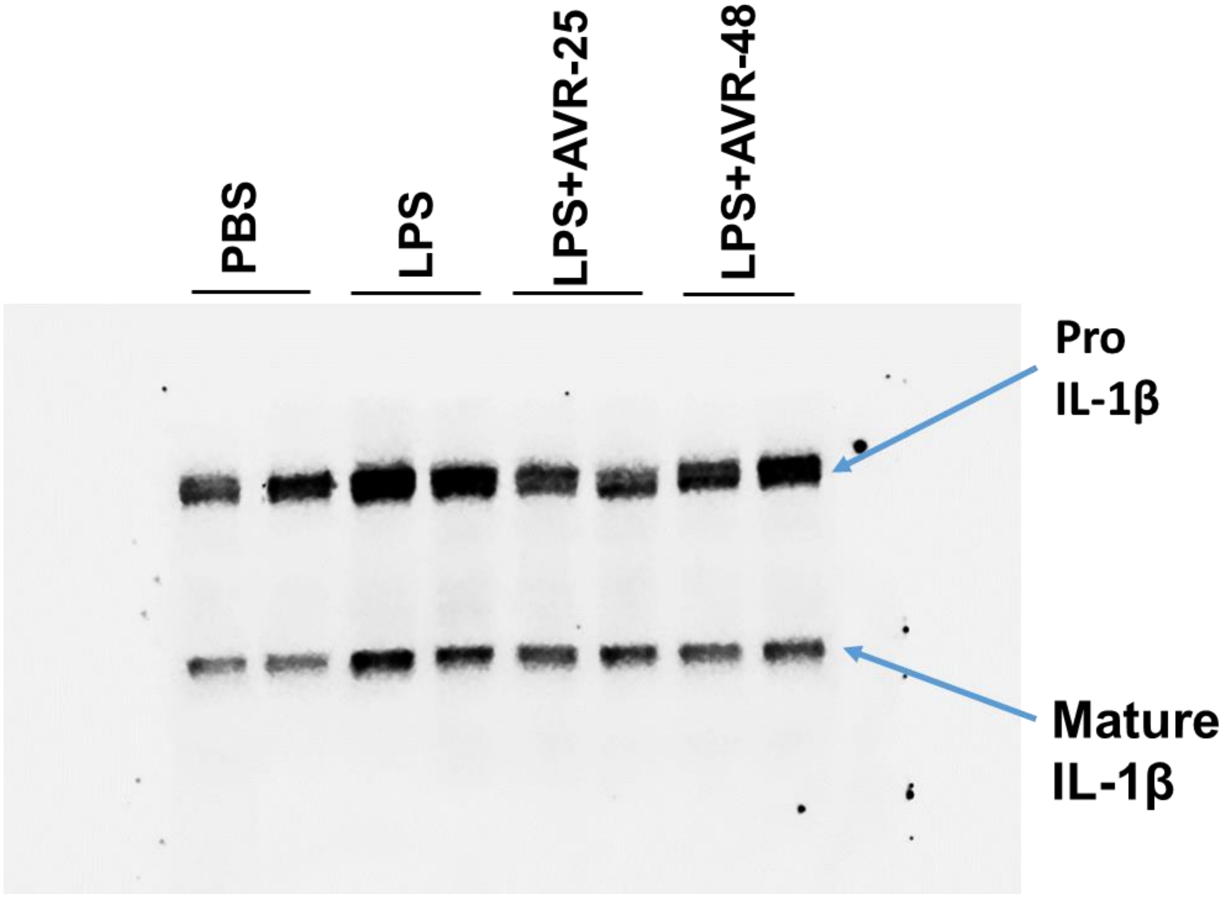

Full uncut immunoblot for Vinculin used in the main **Figure 1F**. Blue box indicates the lanes of Vinculin immunoblots used in the figure.

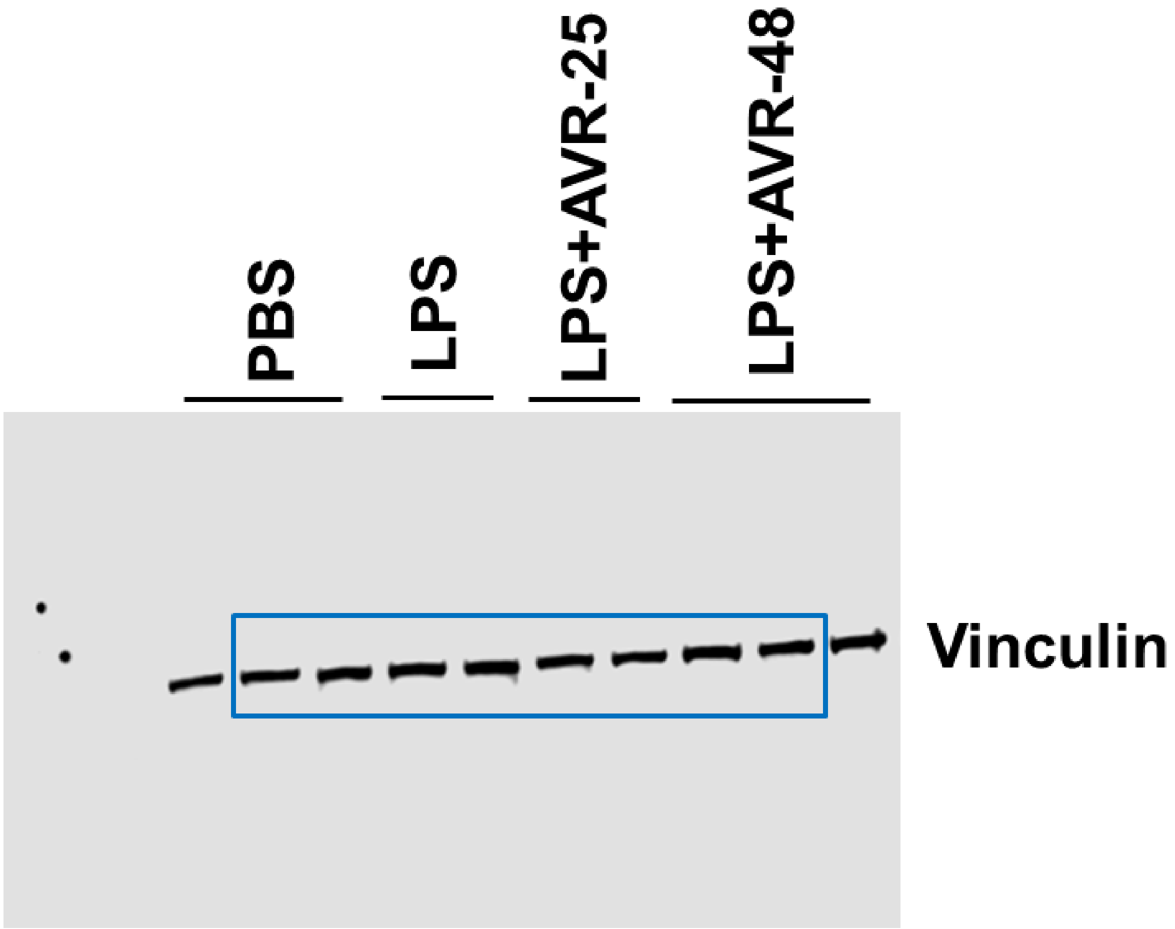

Full uncut immunoblot for IL-6 used in the main **Figure 1L**. Blue box indicates the lanes of IL-6 immunoblots used in the figure.

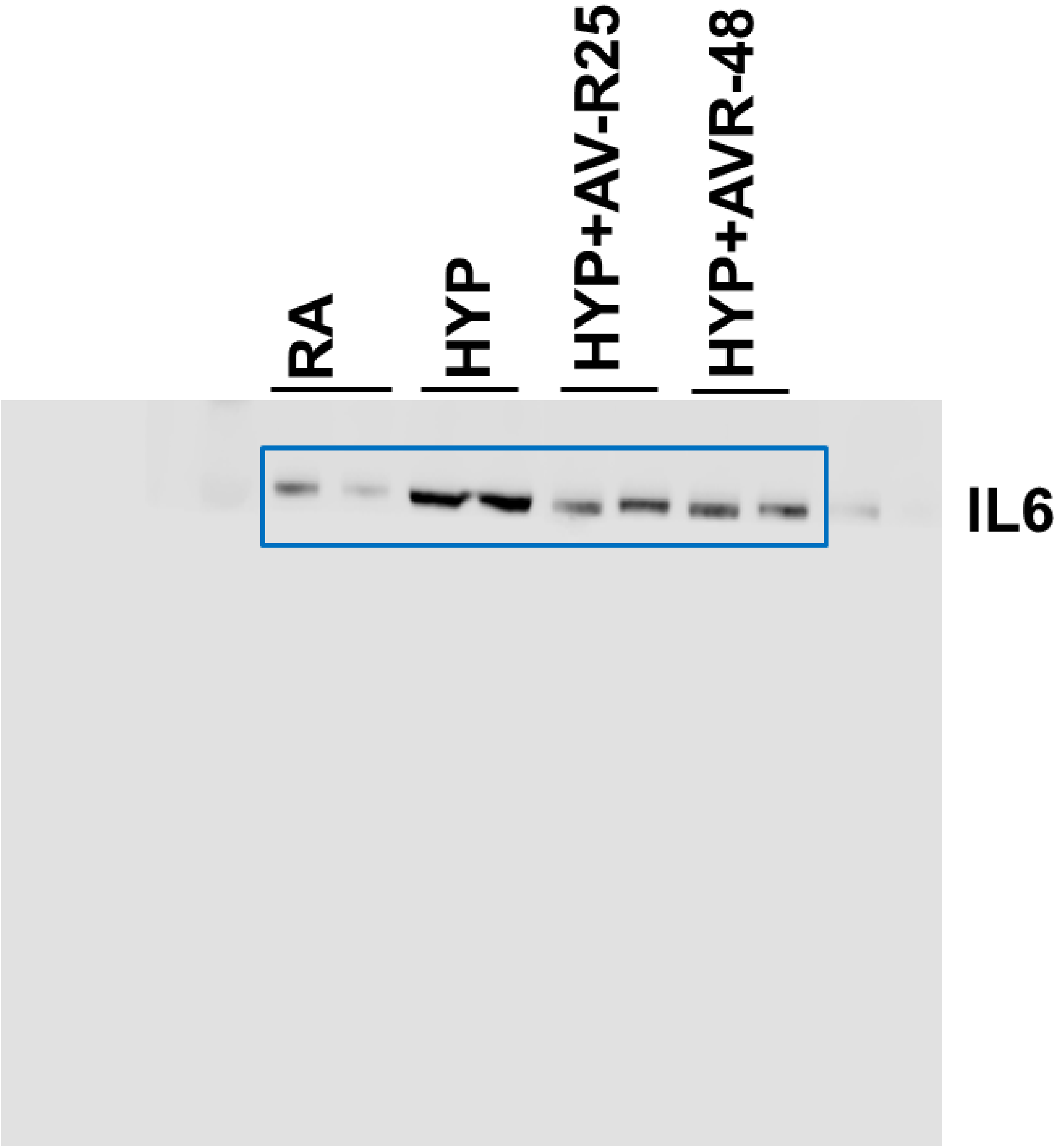

Full uncut immunoblot for IL-1β used in the main **Figure 1L**.

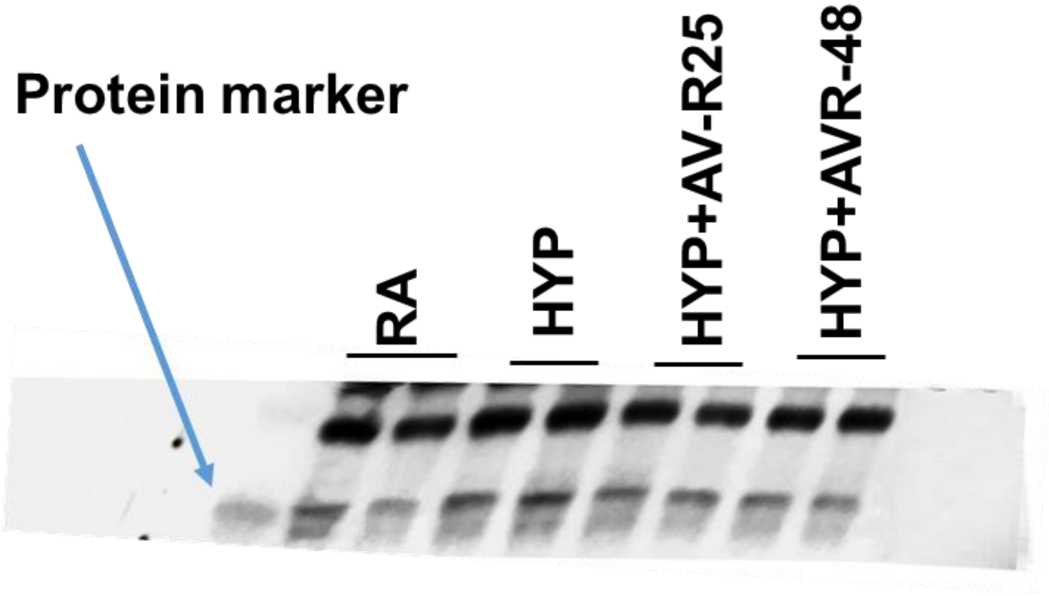

Full uncut immunoblot for Vinculin used in the main **Figure 1L**. Blue box indicates the lanes of Vinculin immunoblots used in the figure.

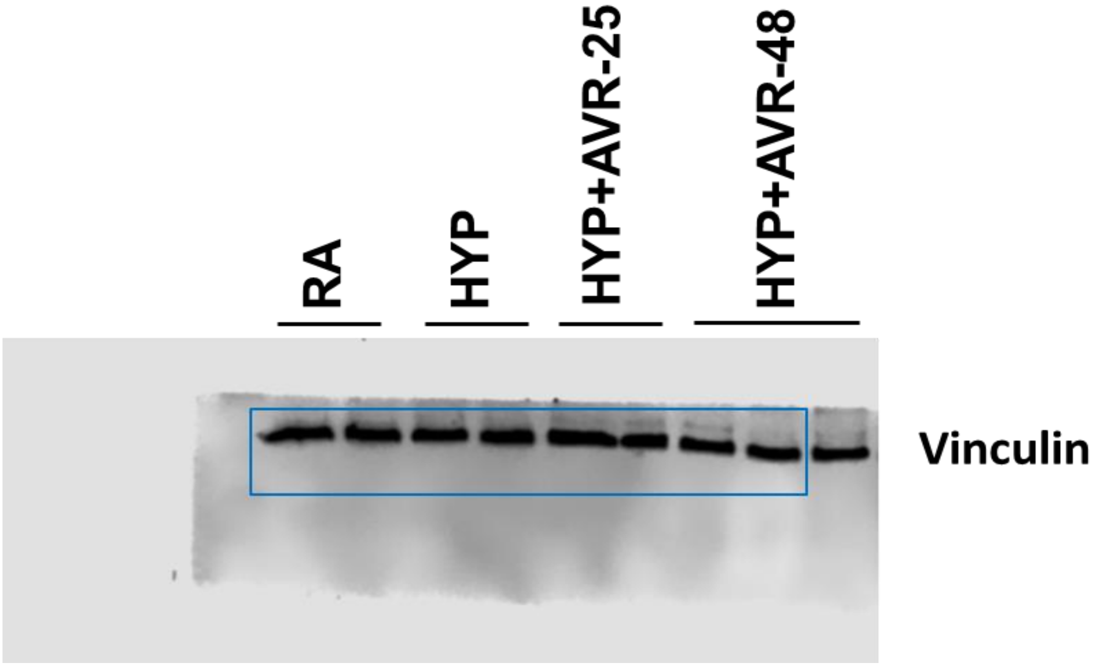

Full uncut immunoblot for ICAM-1 used in the main **Figure 3B**.

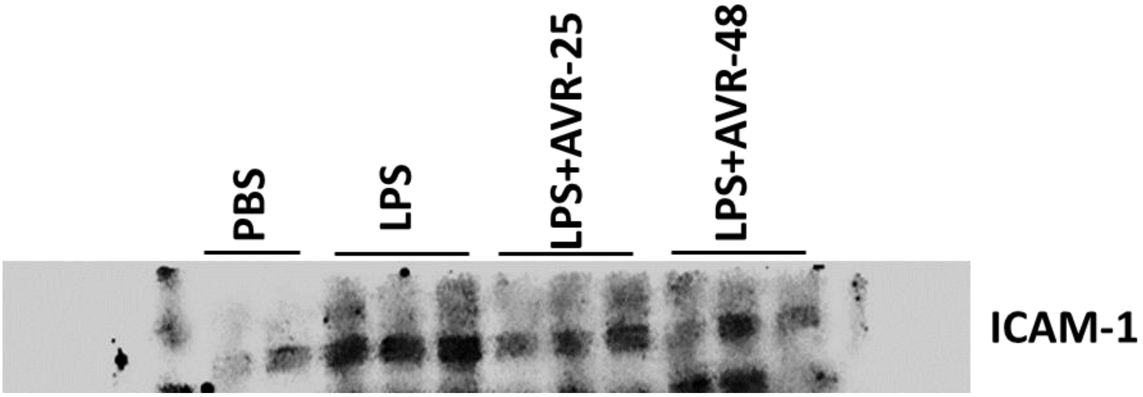

Full uncut immunoblot for VCAM-1 used in the main **Figure 3B**. Blue box indicates the bands of VCAM-1 immunoblots used in the figure.

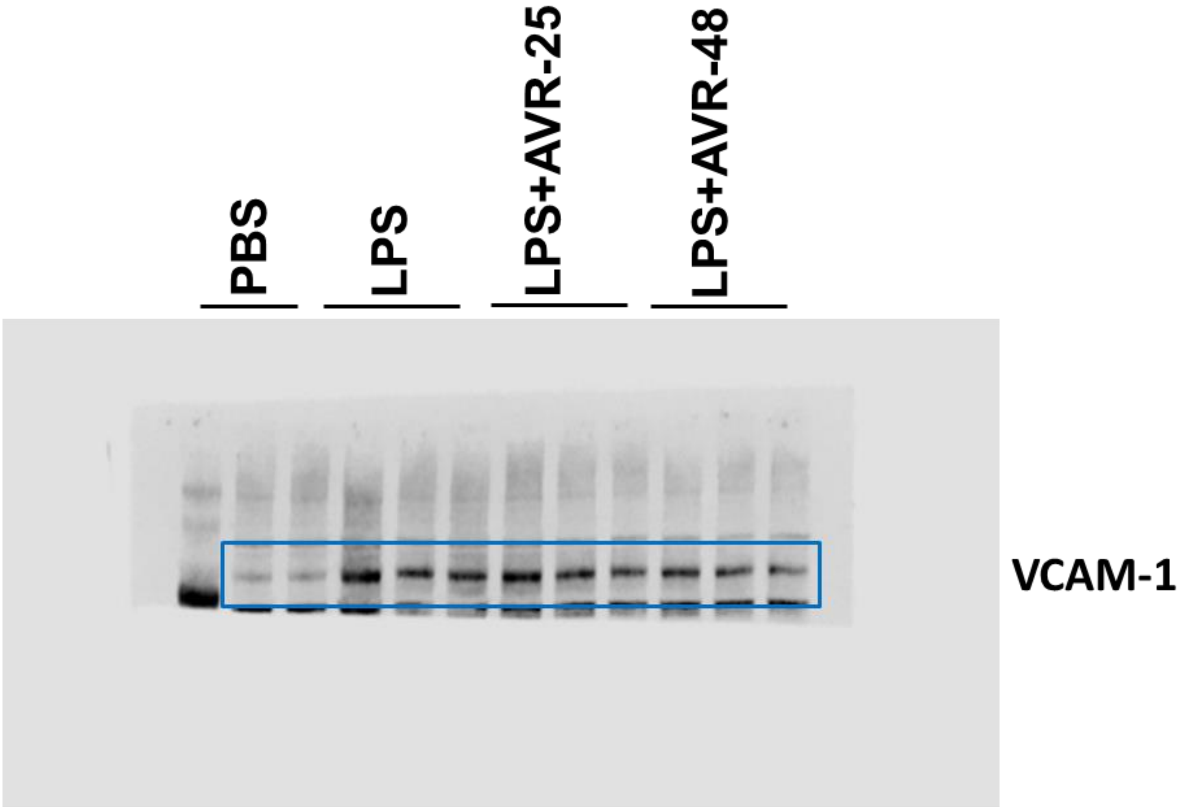

Full uncut immunoblot for E-selectin used in the main **Figure 3B**.

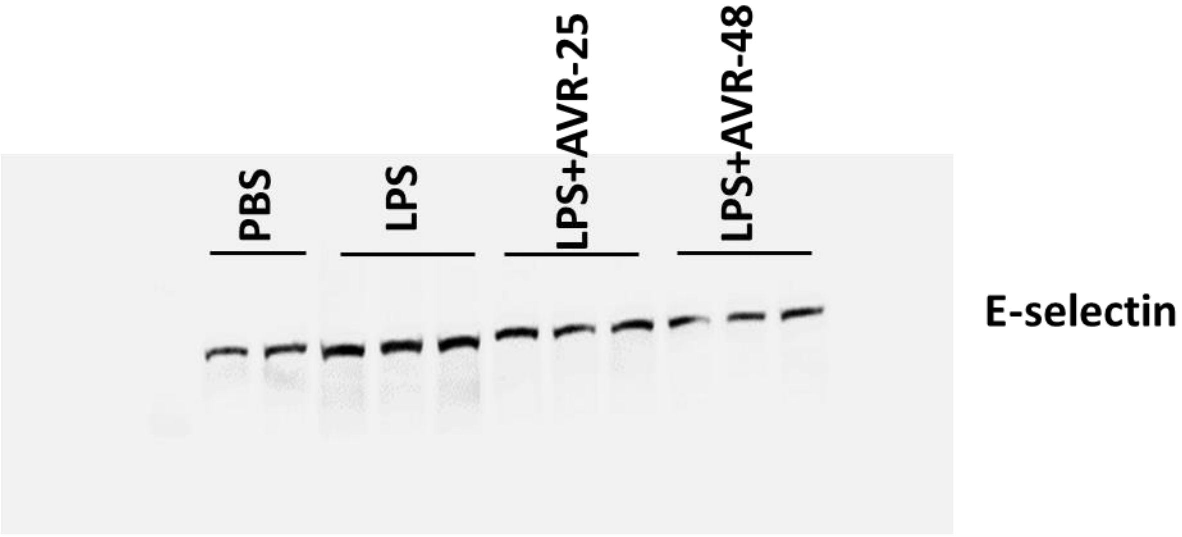

Full uncut immunoblot for Vinculin used in the main **Figure 3B**.

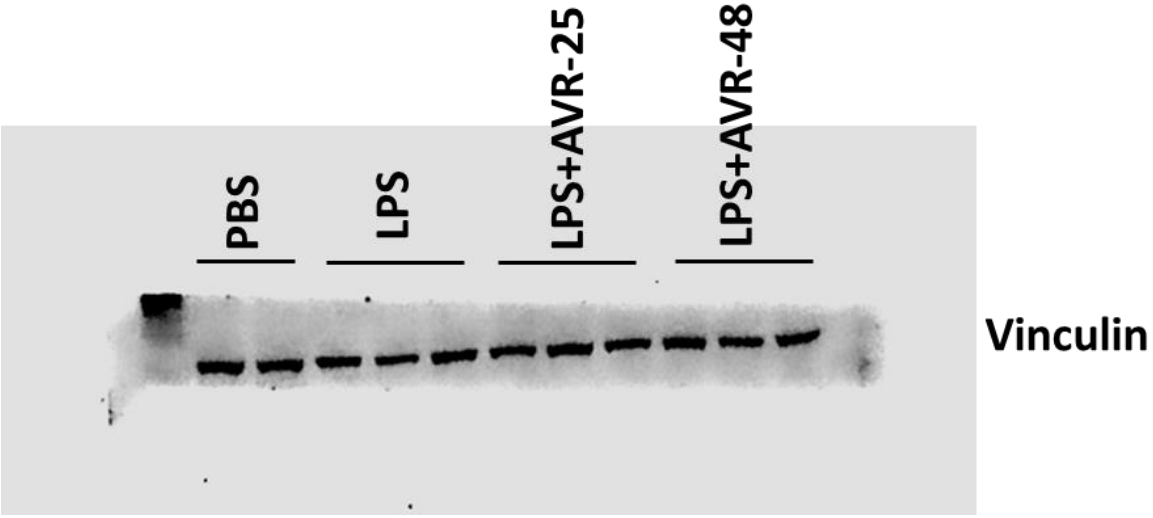

Full uncut immunoblot for ICAM-1 used in the main **Figure 3E**.

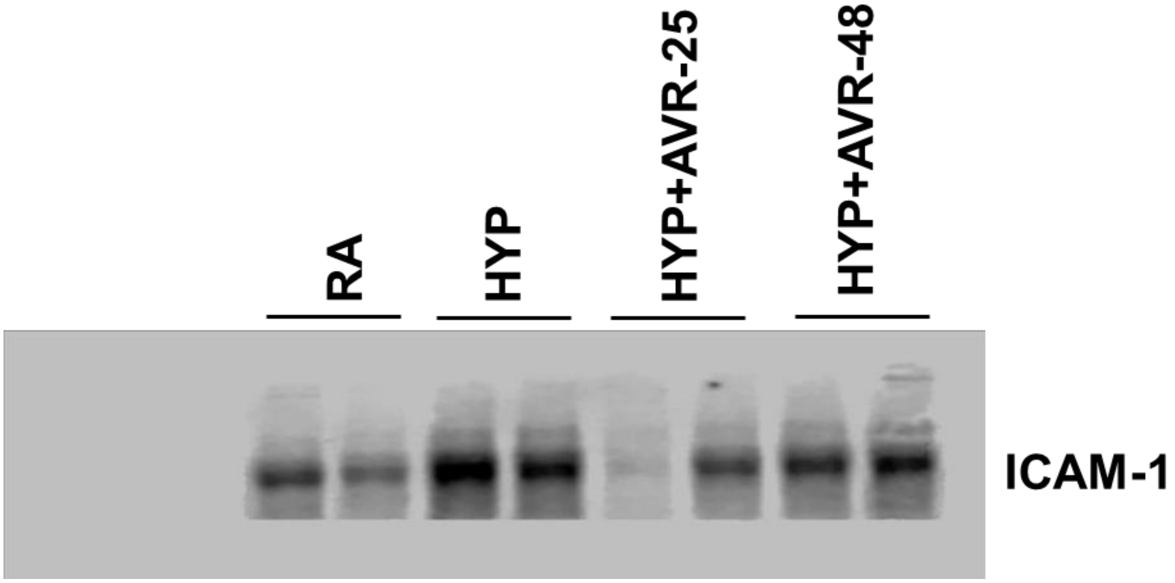

Full uncut immunoblot for VCAM-1 used in the main **Figure 3E**. Blue box indicates the bands of VCAM-1 immunoblots used in the figure.

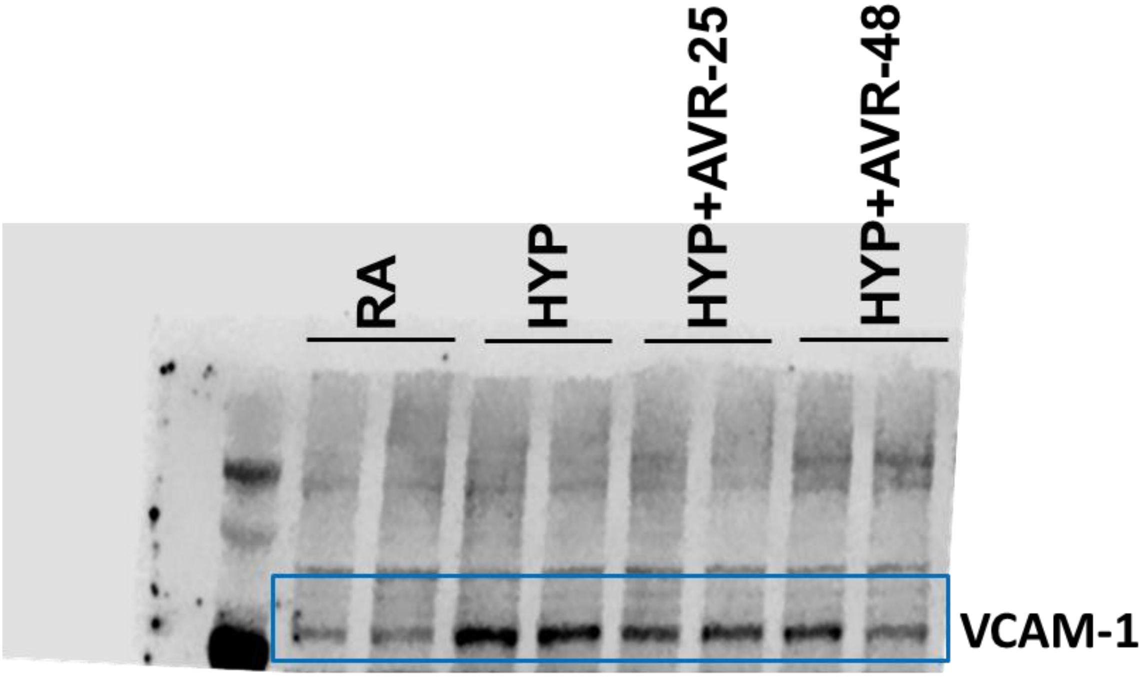

Full uncut immunoblot for Vinculin used in the main **Figure 3E**.

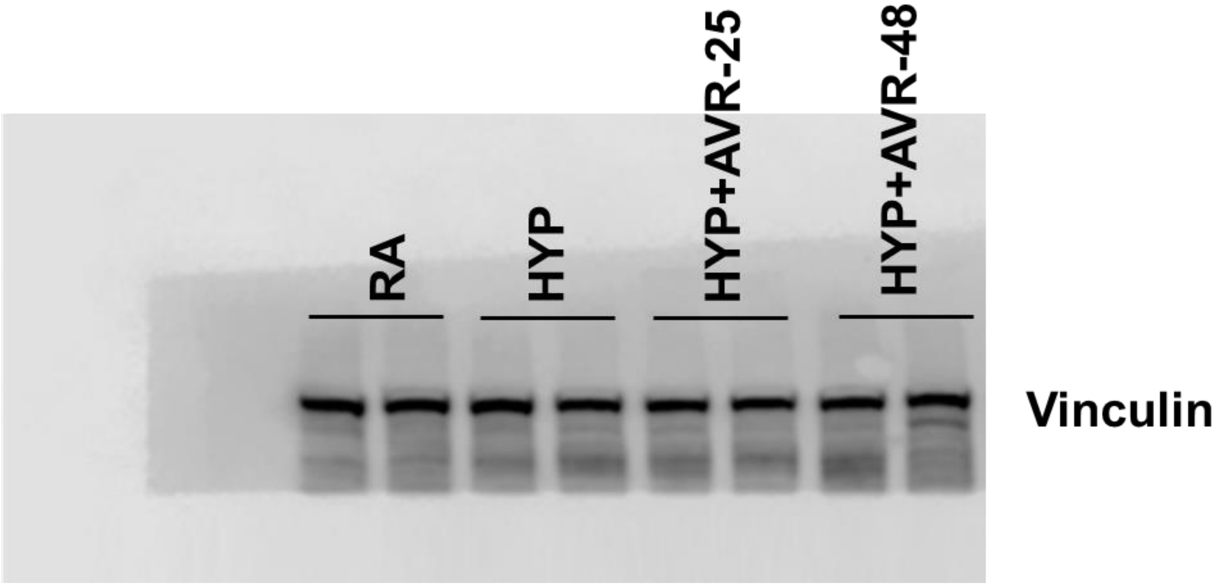

Full uncut immunoblot for p-Src used in the main **Figure 4A**. Blue box indicates the lanes of p-Src immunoblots used in the figure.

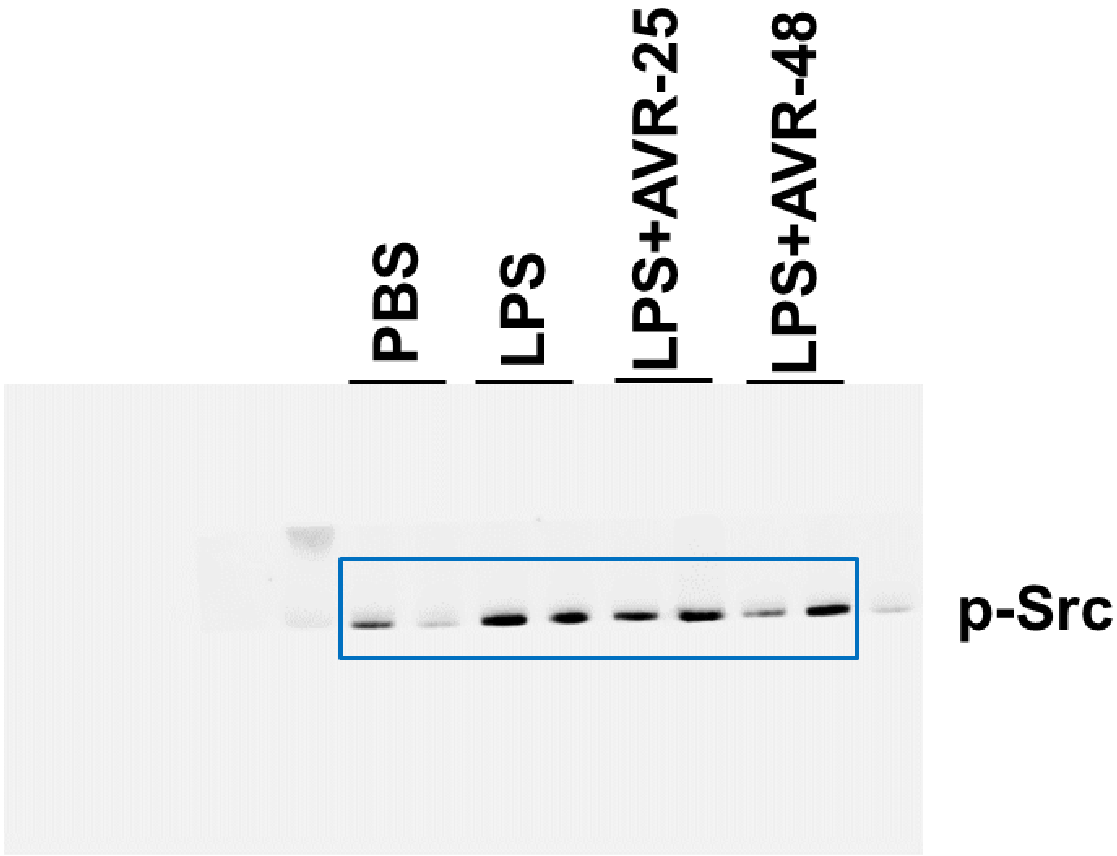

Full uncut immunoblot for Src used in the main **Figure 4A**. Blue box indicates the lanes of Src immunoblots used in the figure.

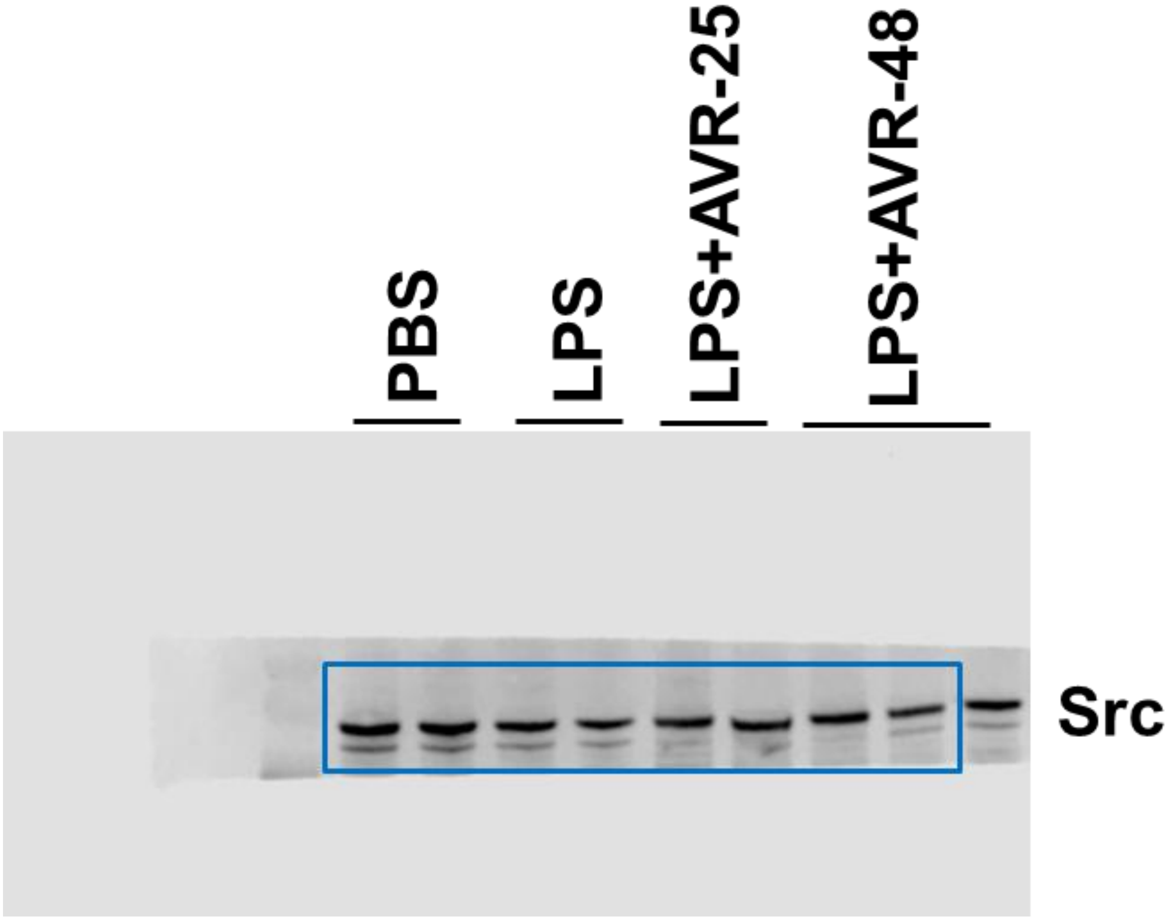

Full uncut immunoblot for VE-cadherin used in the main **Figure 4A**. Blue box indicates the lanes of p-Src immunoblots used in the figure.

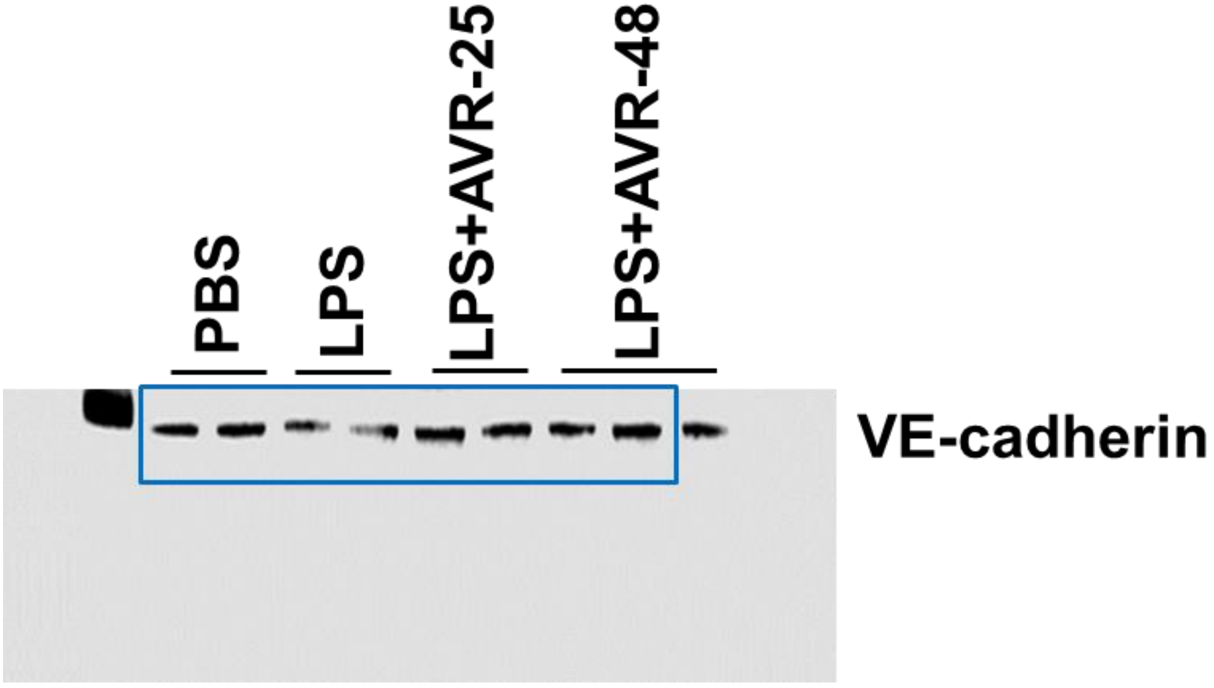

Full uncut immunoblot for β-Catenin used in the main **Figure 4A**. Blue box indicates the lanes of β-Catenin immunoblots used in the figure.

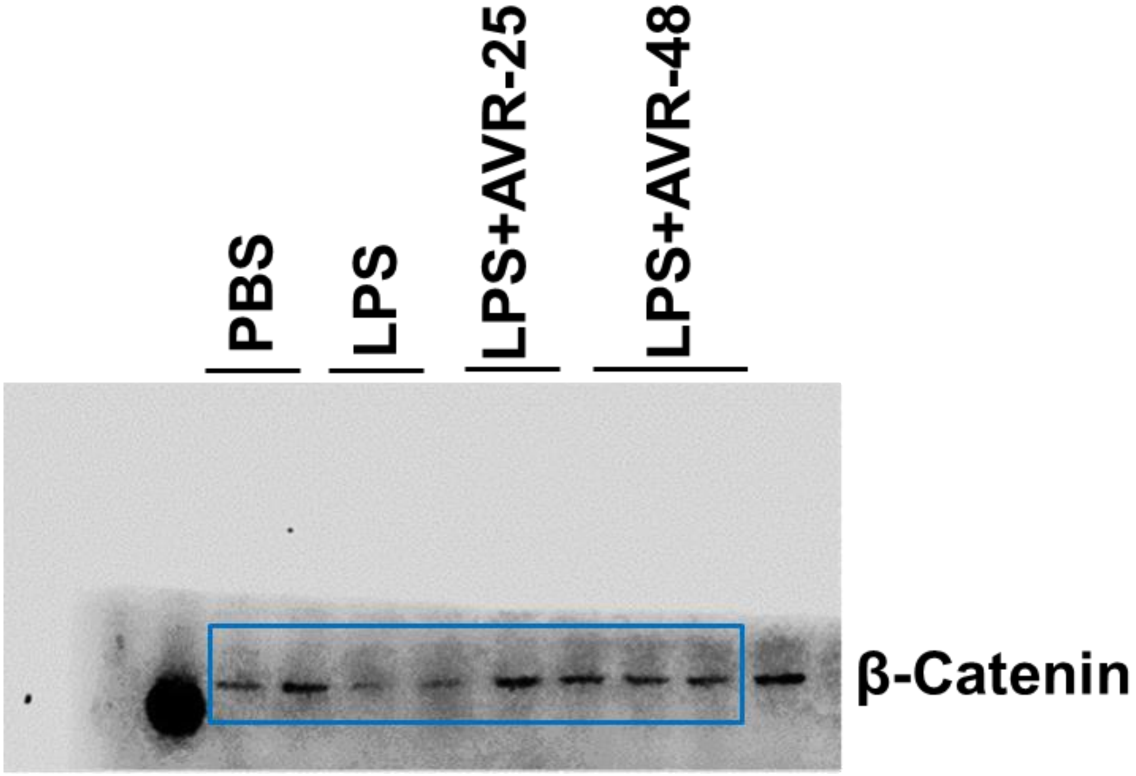

Full uncut immunoblot for Vinculin used in the main **Figure 4A**. Blue box indicates the lanes of Vinculin immunoblots used in the figure.

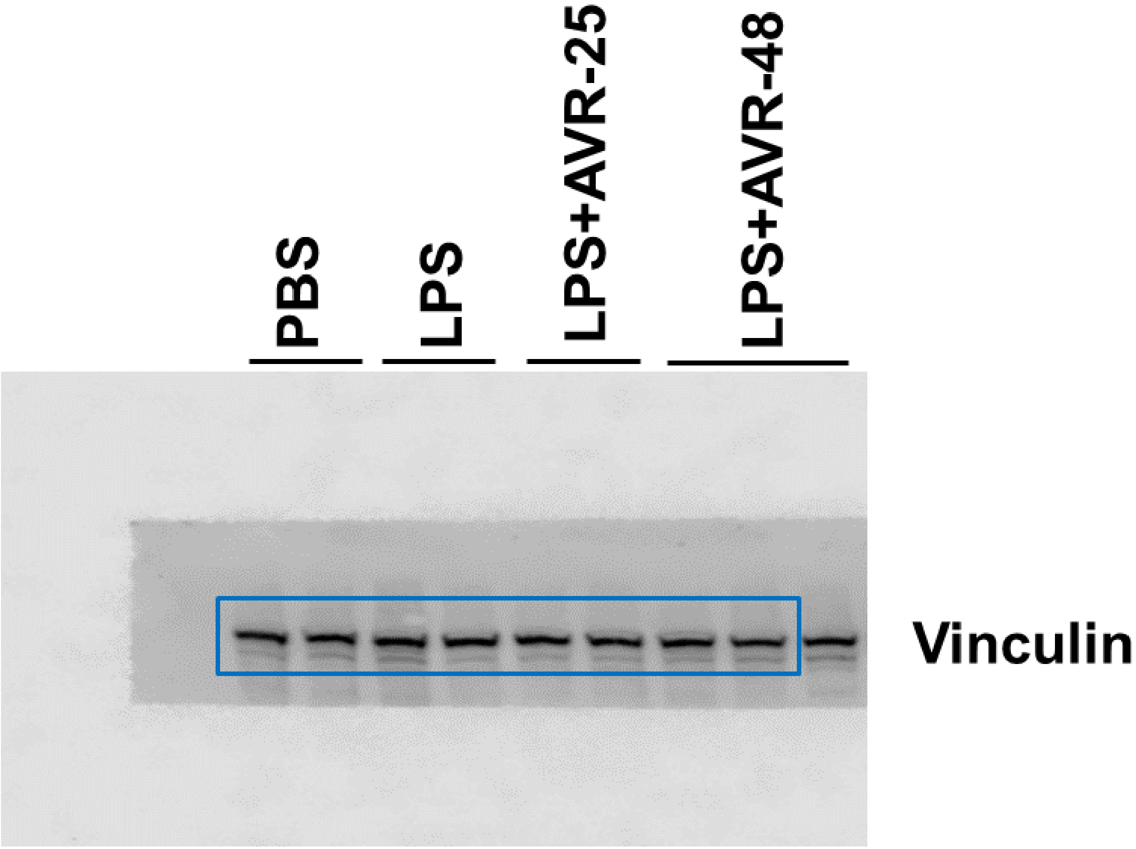

Full uncut immunoblot for p-Src used in the main **Figure 4C**.

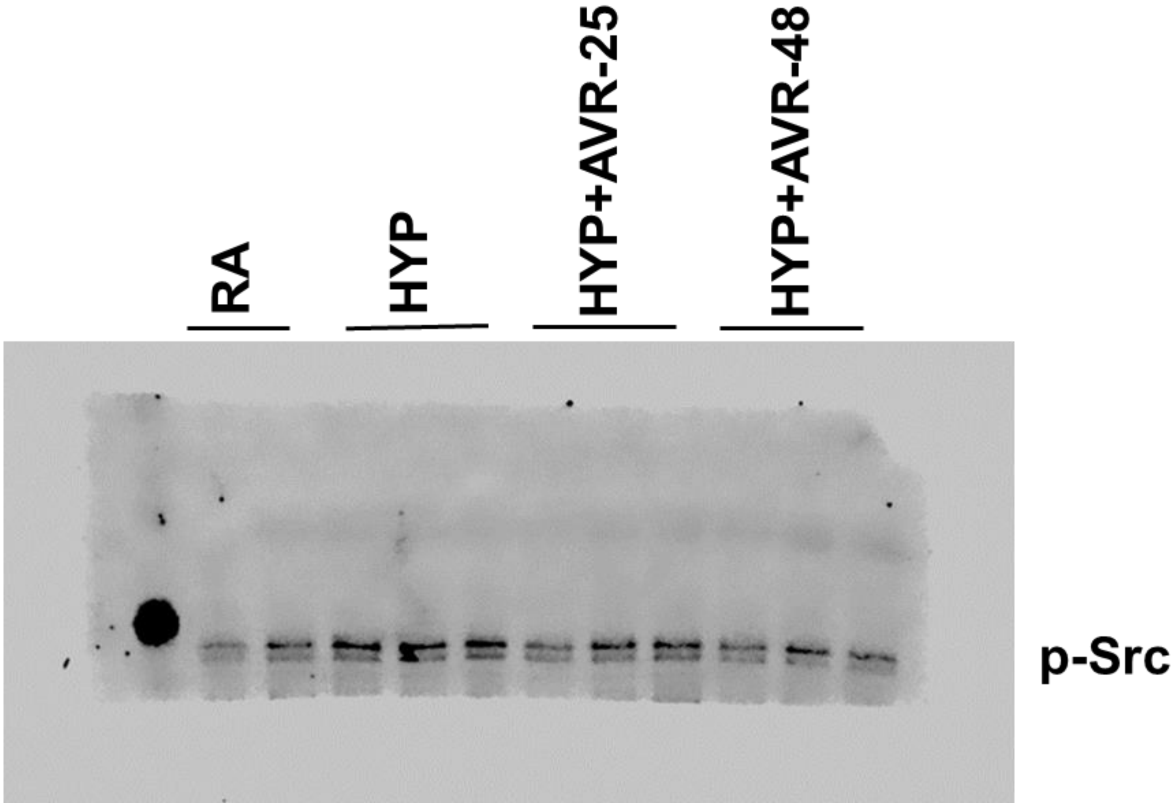

Full uncut immunoblot for Src used in the main **Figure 4C**.

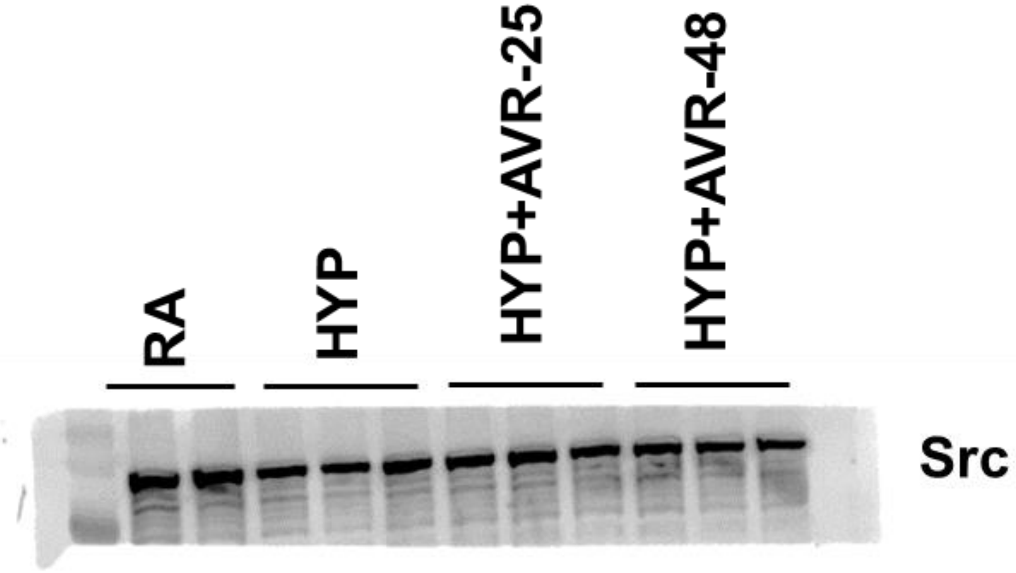

Full uncut immunoblot for β-Catenin used in the main **Figure 4C**.

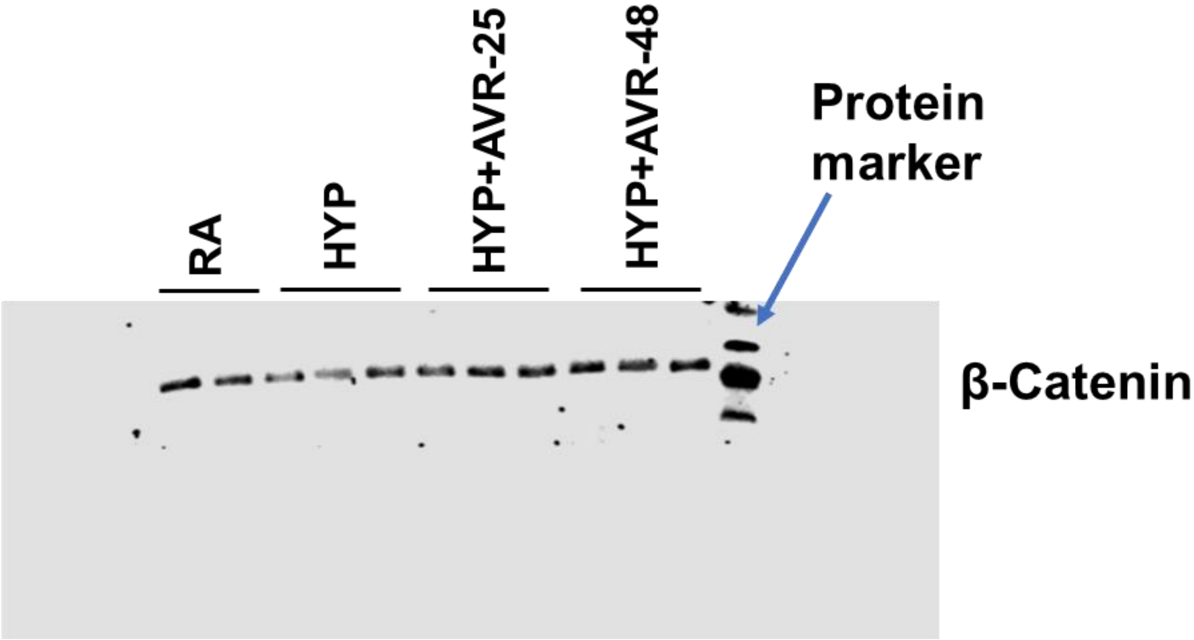

Full uncut immunoblot for VE-cadherin used in the main **Figure 4C**.

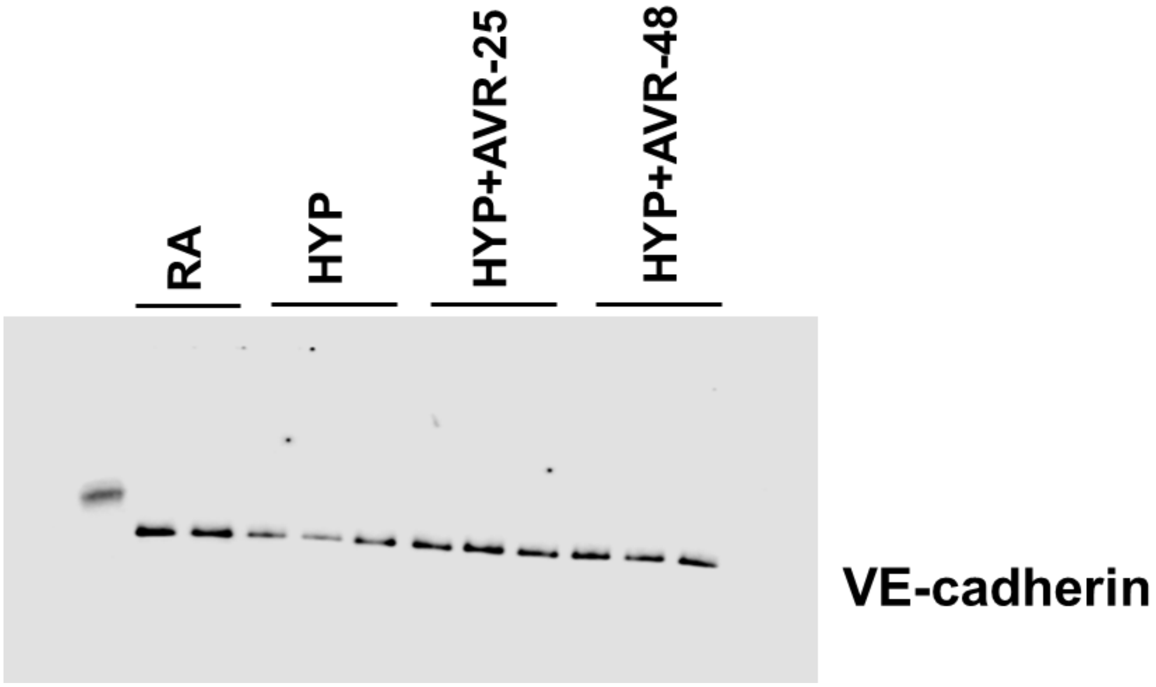

Full uncut immunoblot for Vinculin used in the main **Figure 4C**.

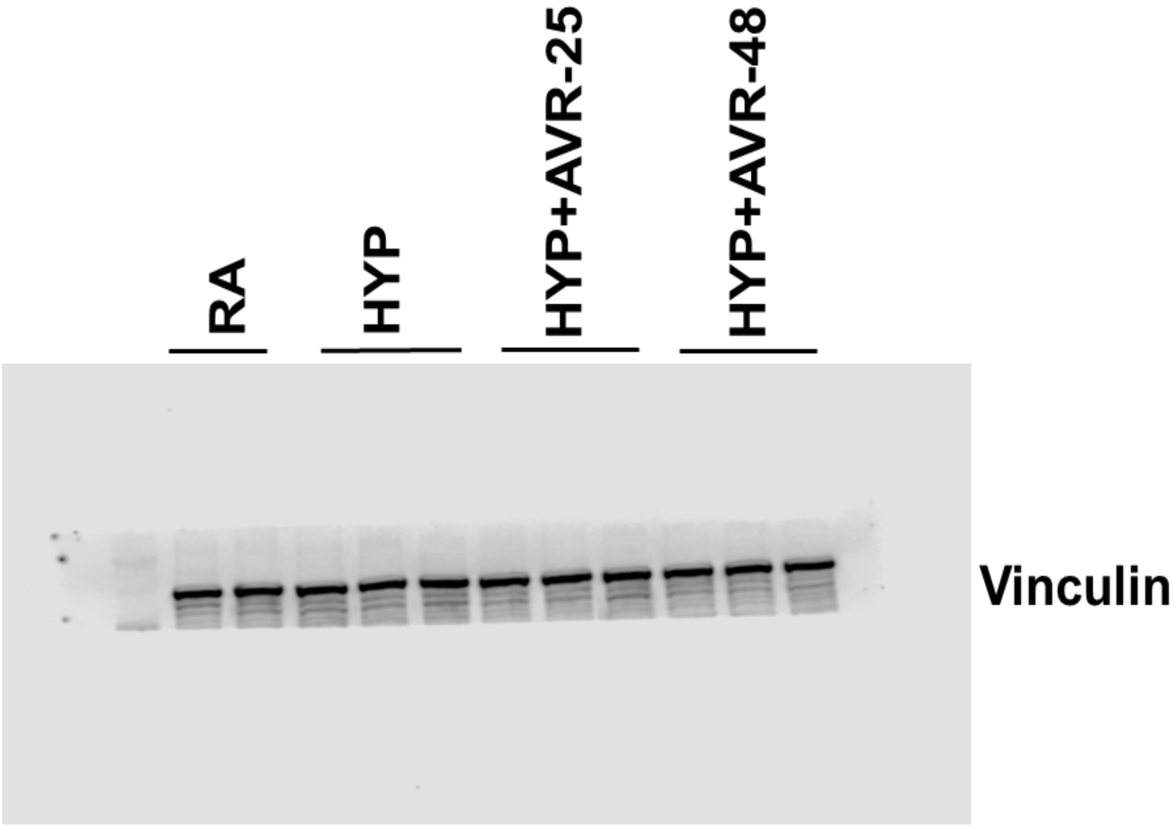

Full uncut immunoblot for cleaved caspase3 used in the main **Figure 5A**. Blue box indicates the lanes of cleaved caspase-3 immunoblots used in the figure.

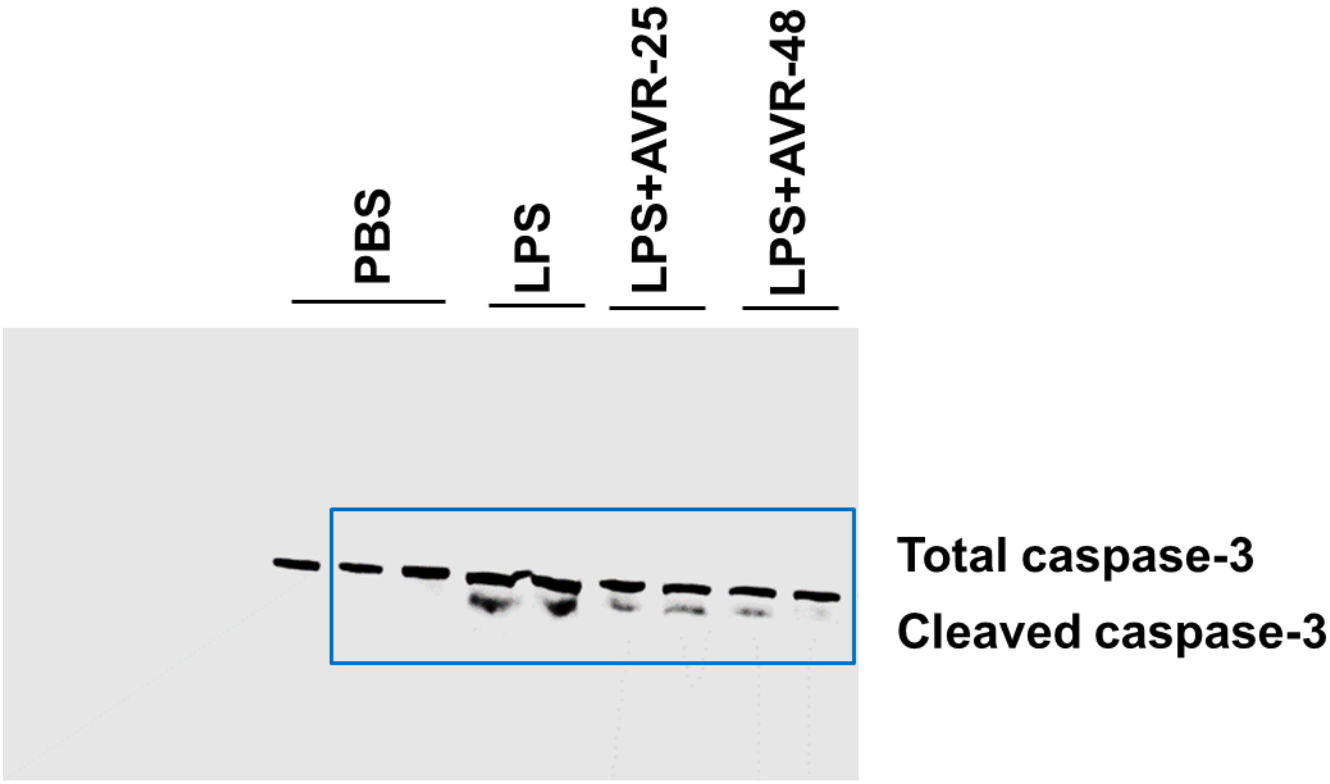

Full uncut immunoblot for vinculin used in the main **Figure 5A**. Blue box indicates the lanes of vinculin immunoblots used in the figure.

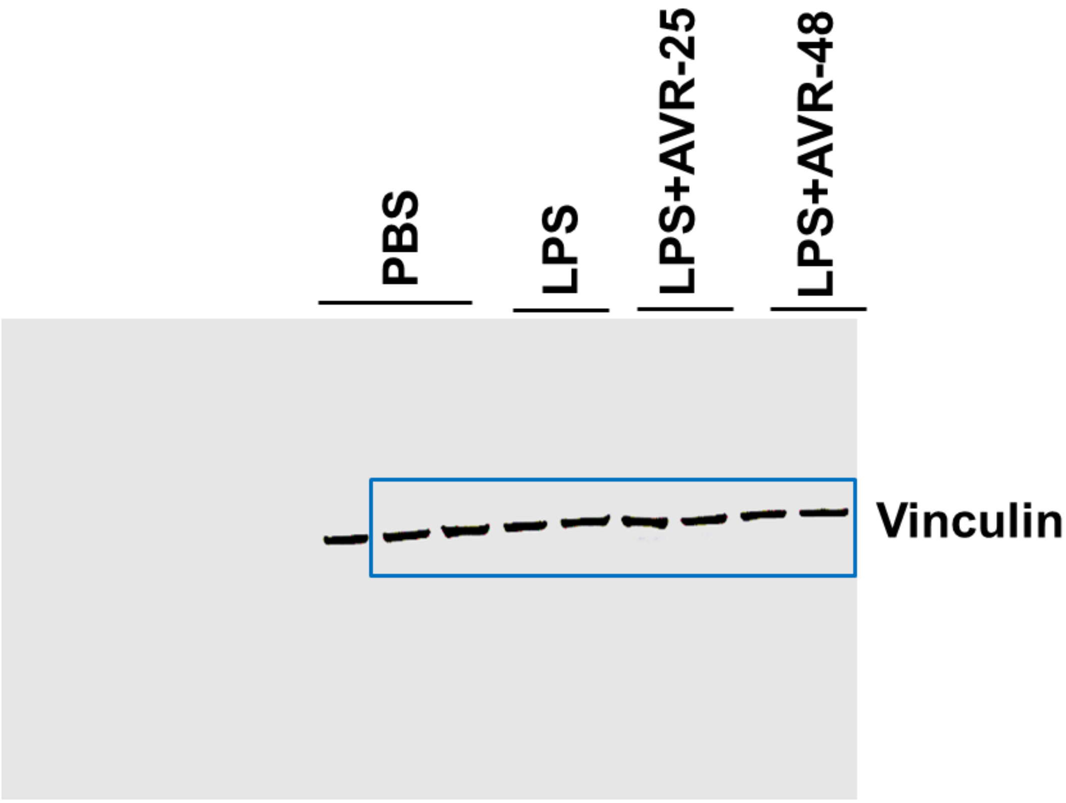

Full uncut immunoblot for cleaved caspase3 used in the main **Figure 5C**. Blue box indicates the lanes of cleaved caspase-3 immunoblots used in the figure.

.

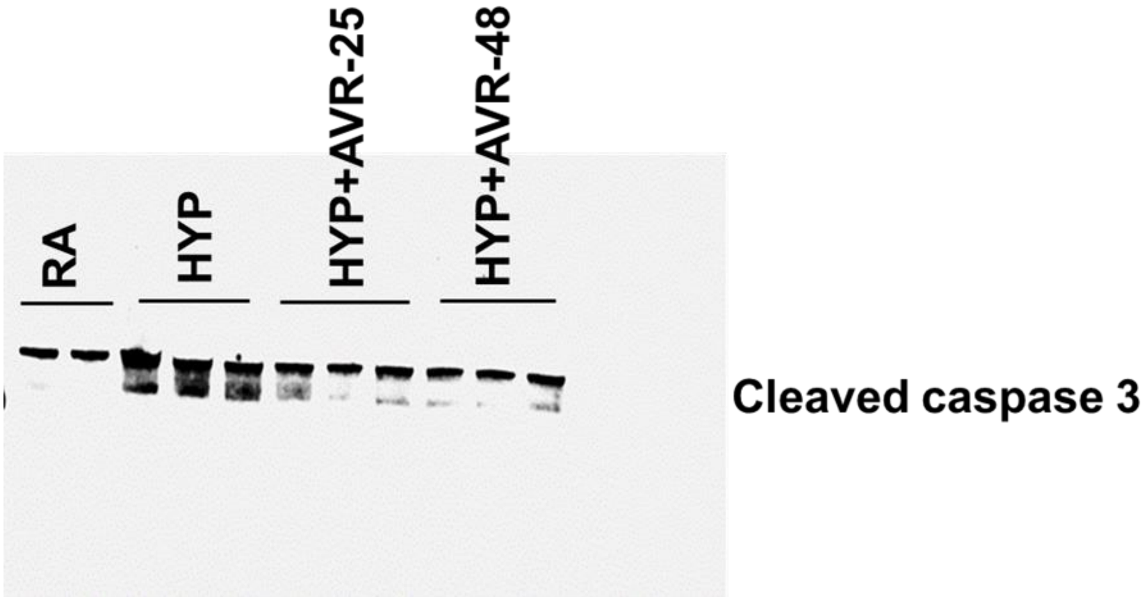

Full uncut immunoblot for GAPDH used in the main **Figure 5C**.

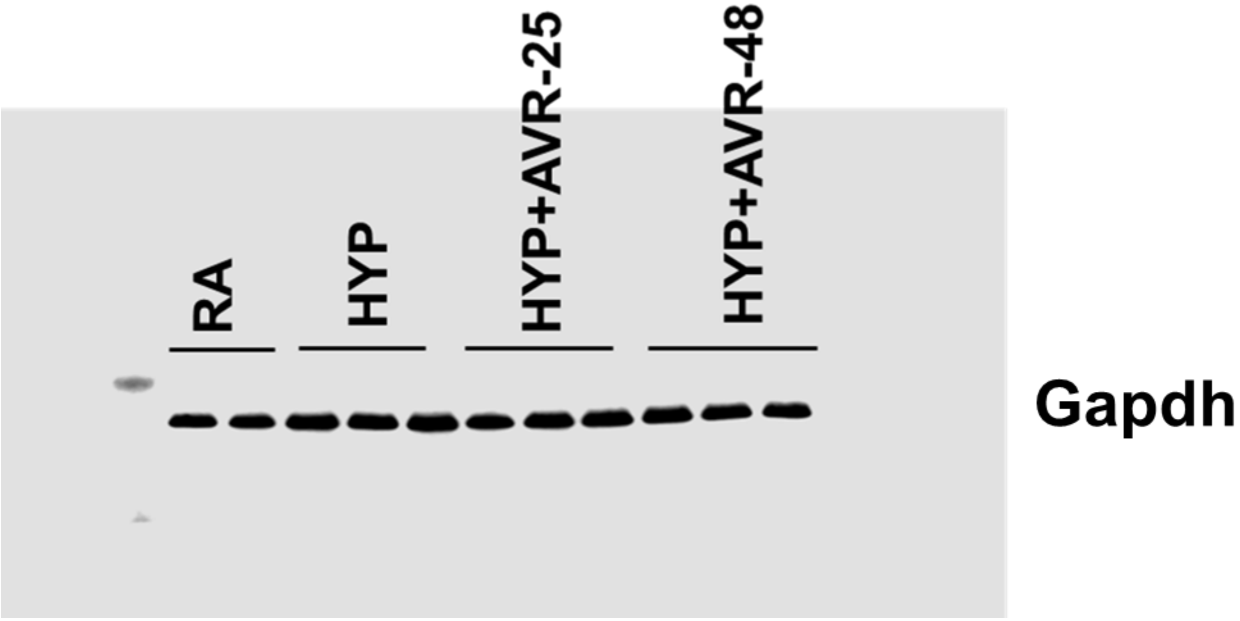

Full uncut image of TUNEL staining in lung used in **Figure 5E**

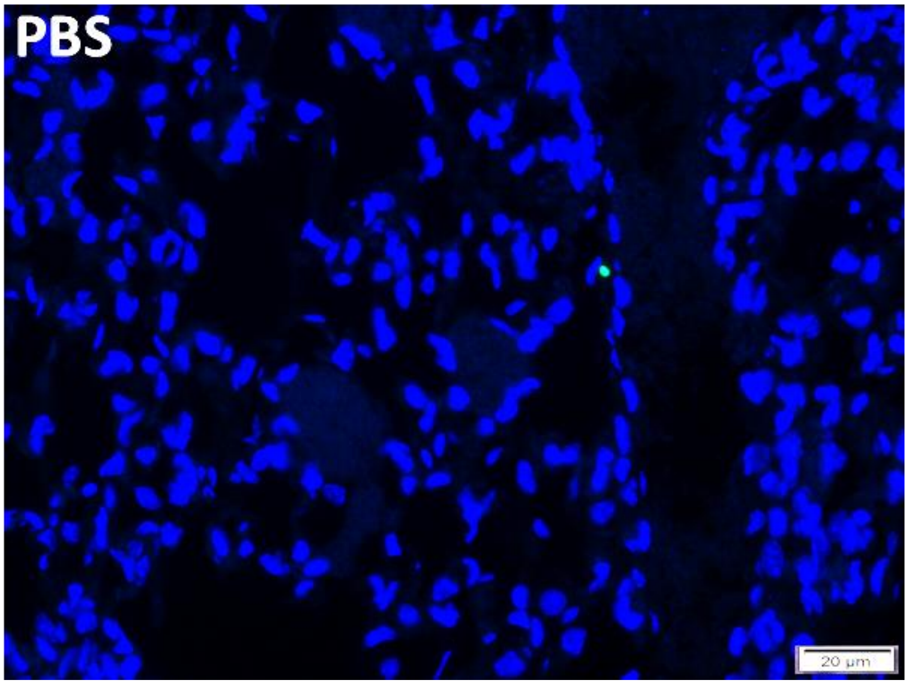

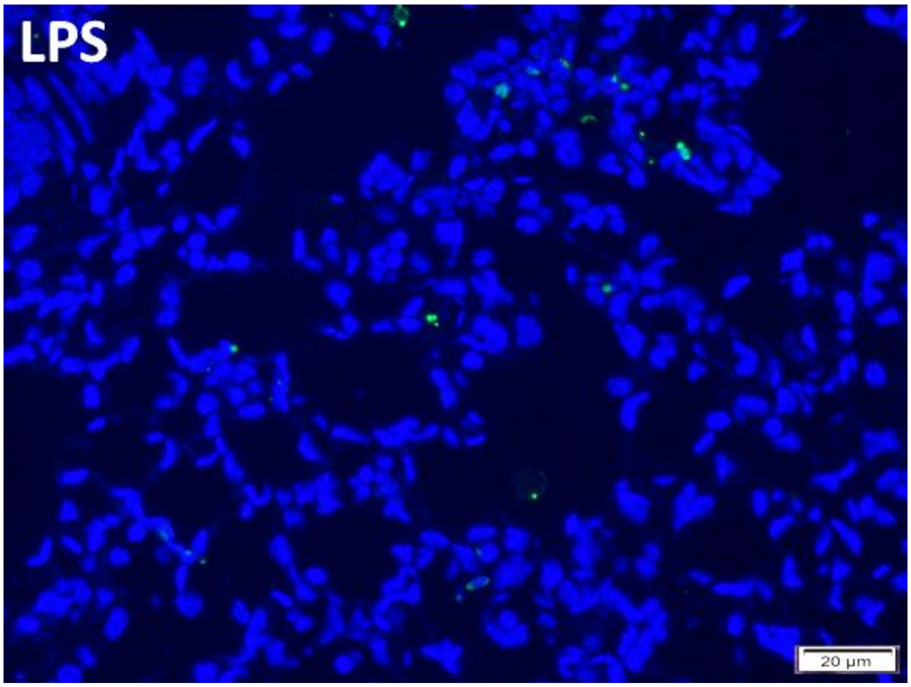

Full uncut image of TUNEL staining in lung used in **Figure 5E**

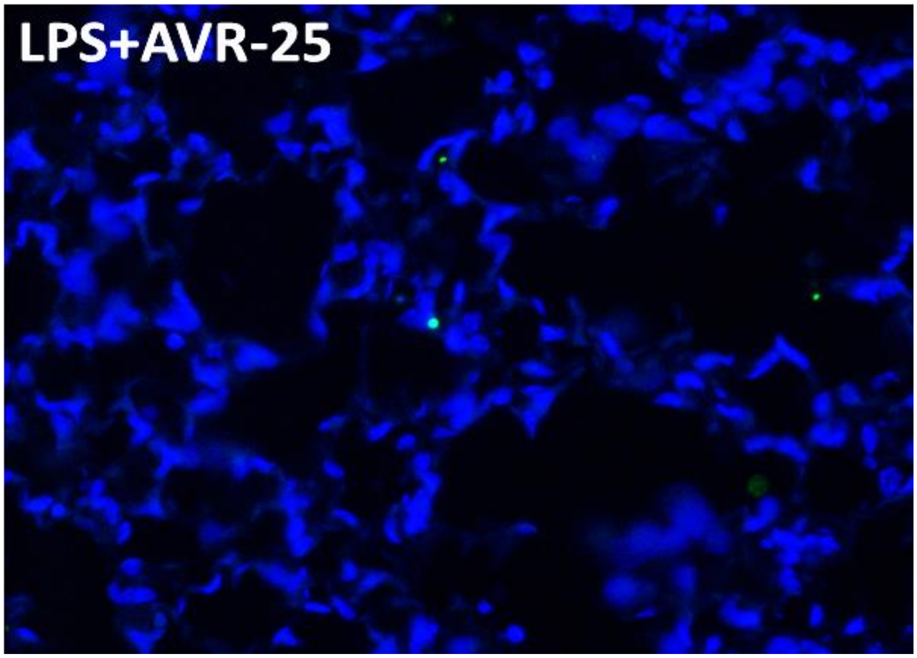

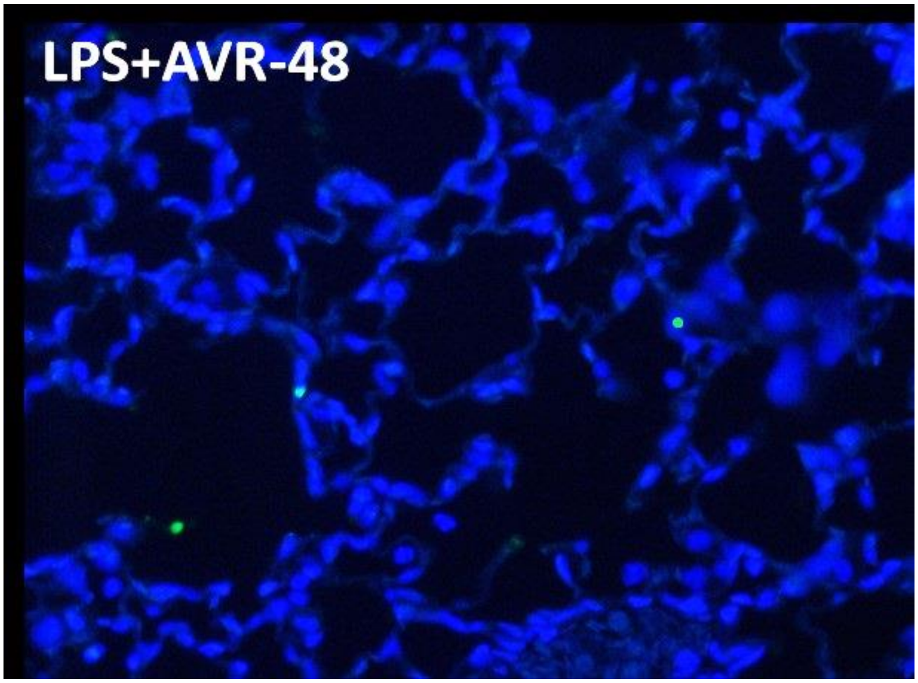

Full uncut image of lungs H-E staining used in **Figure 6A**

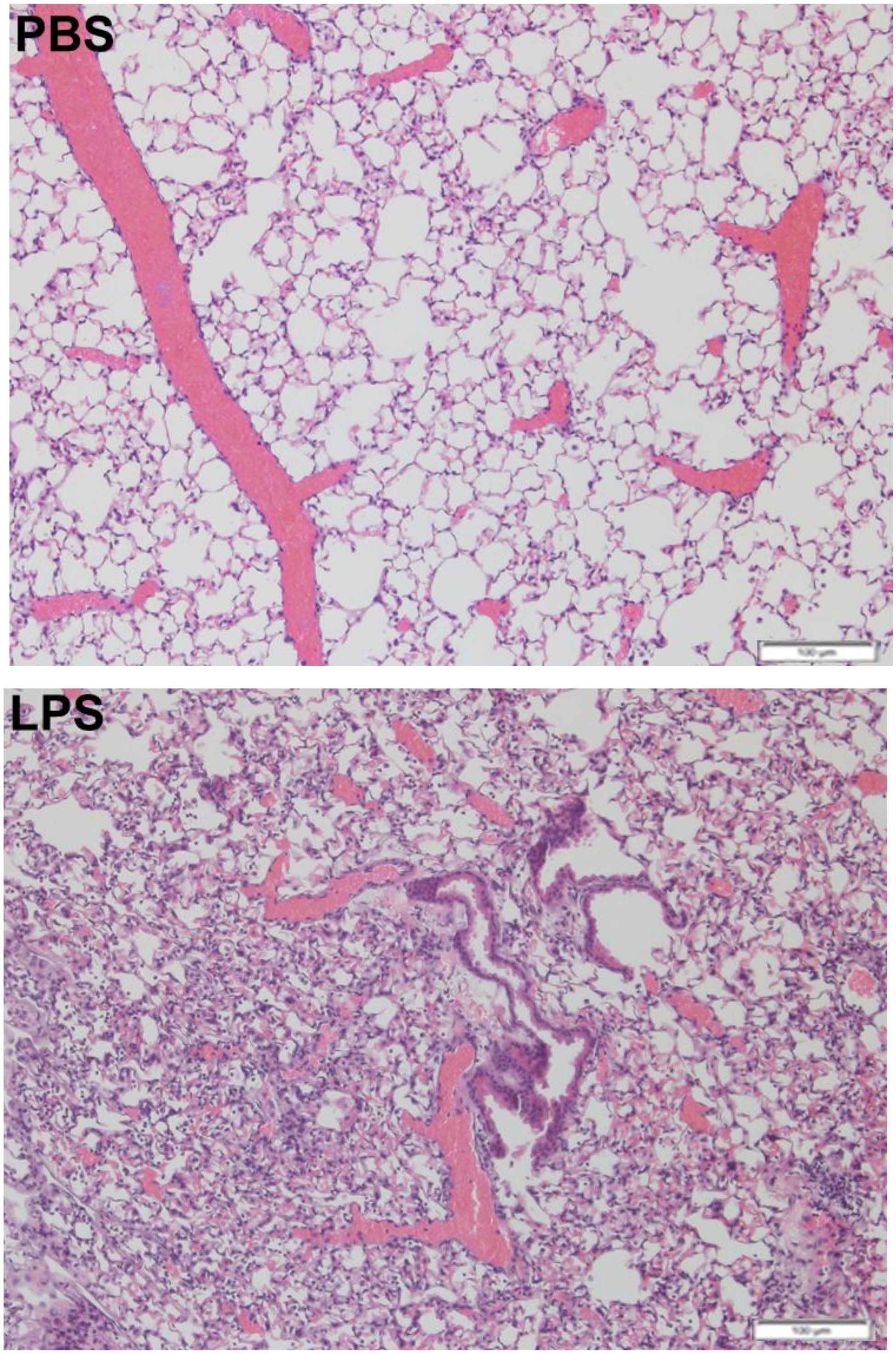

Full uncut image of lungs H-E staining used in **Figure 6A**

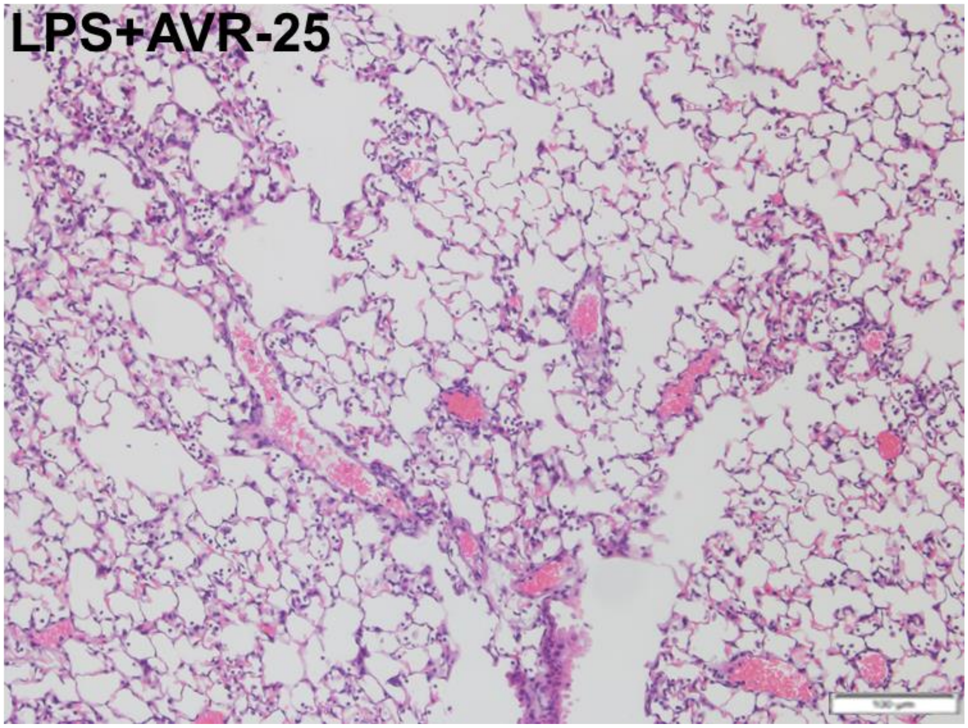

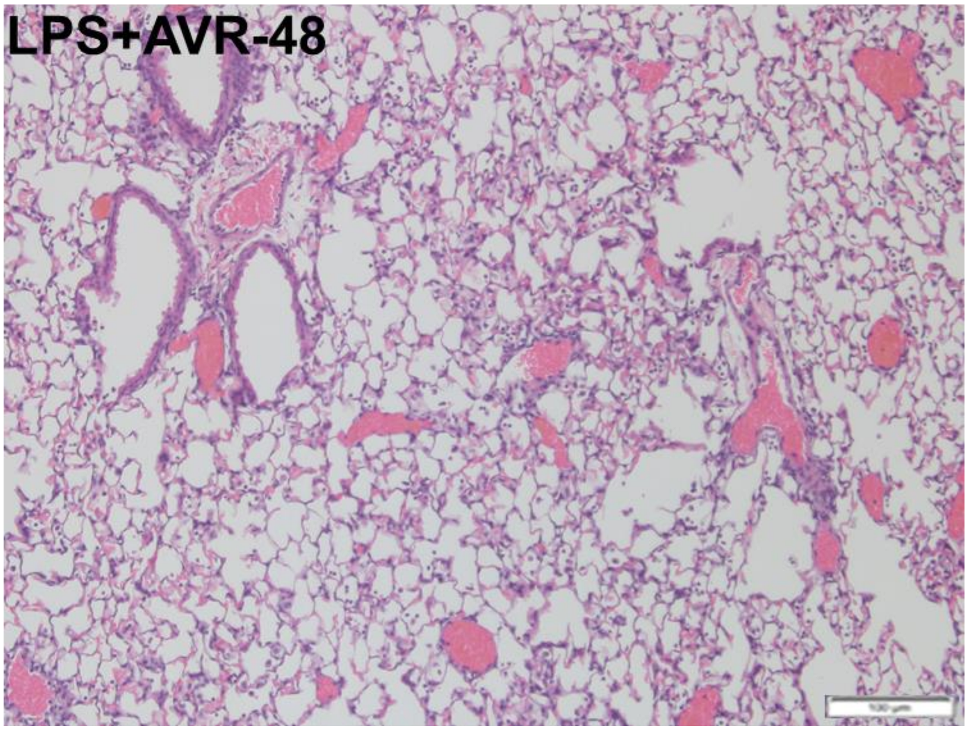

Full uncut image of lungs H-E staining used in **Figure 6C**

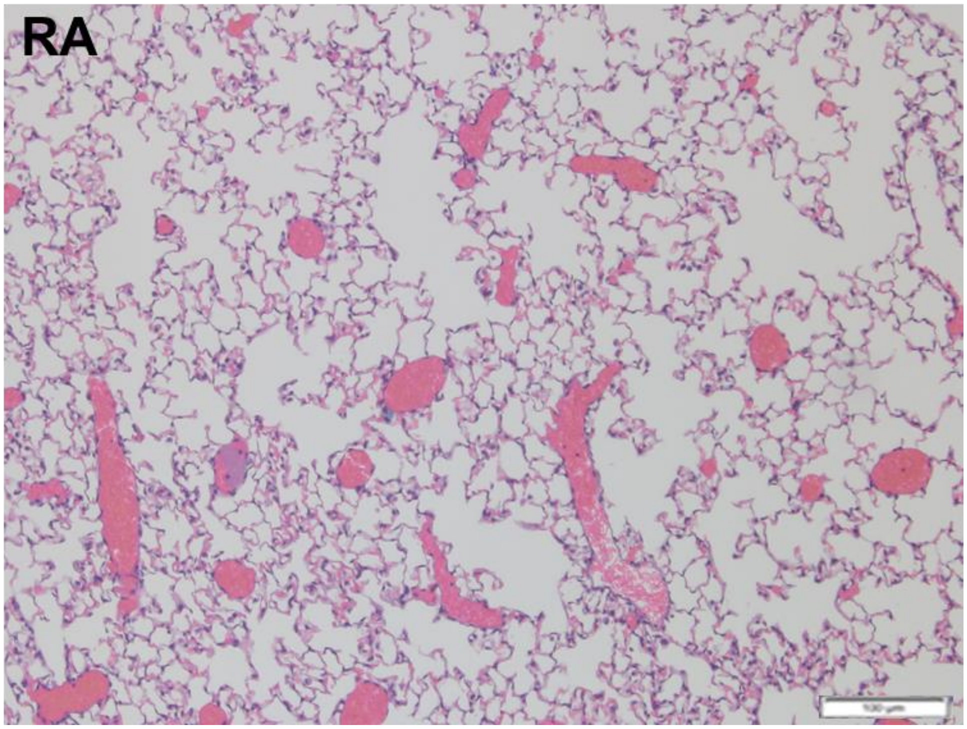

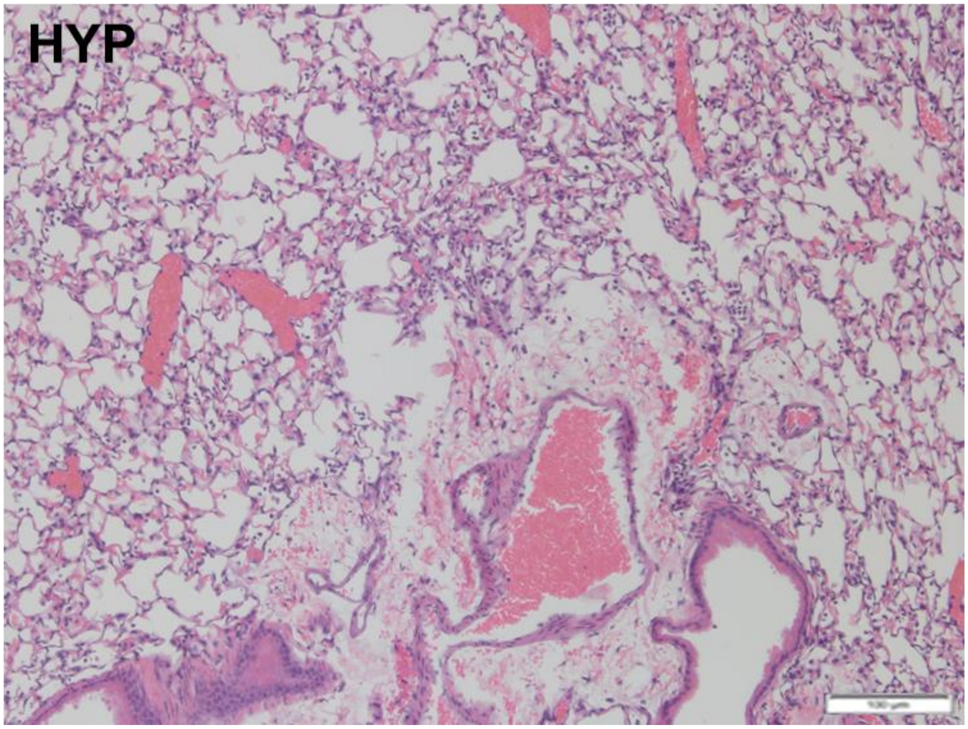

Full uncut image of lungs H-E staining used in **Figure 6C**

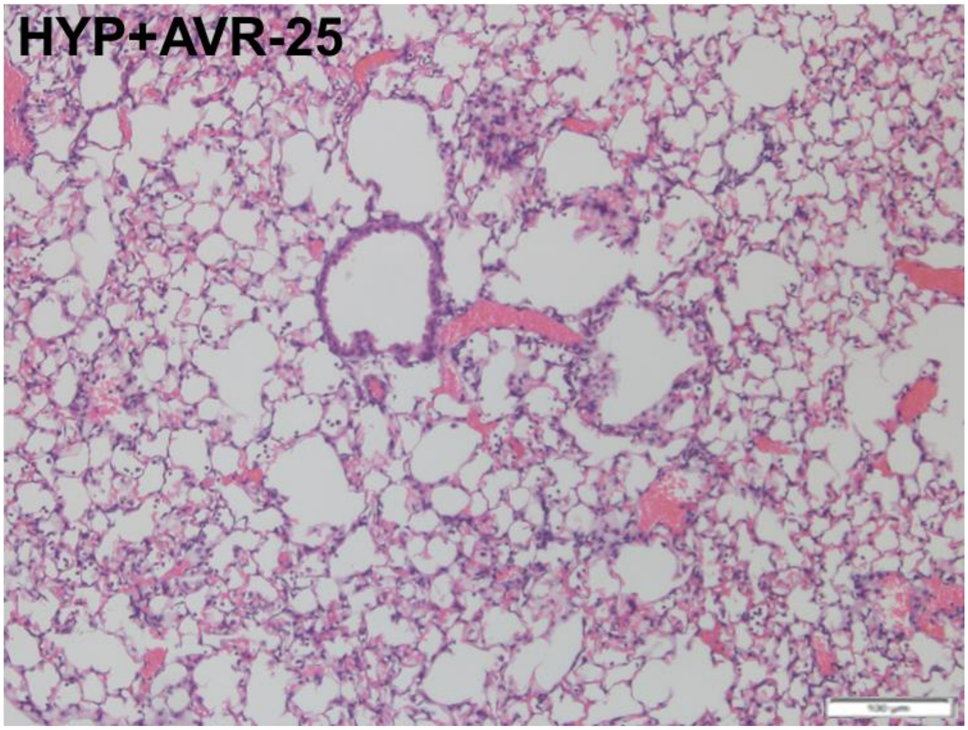

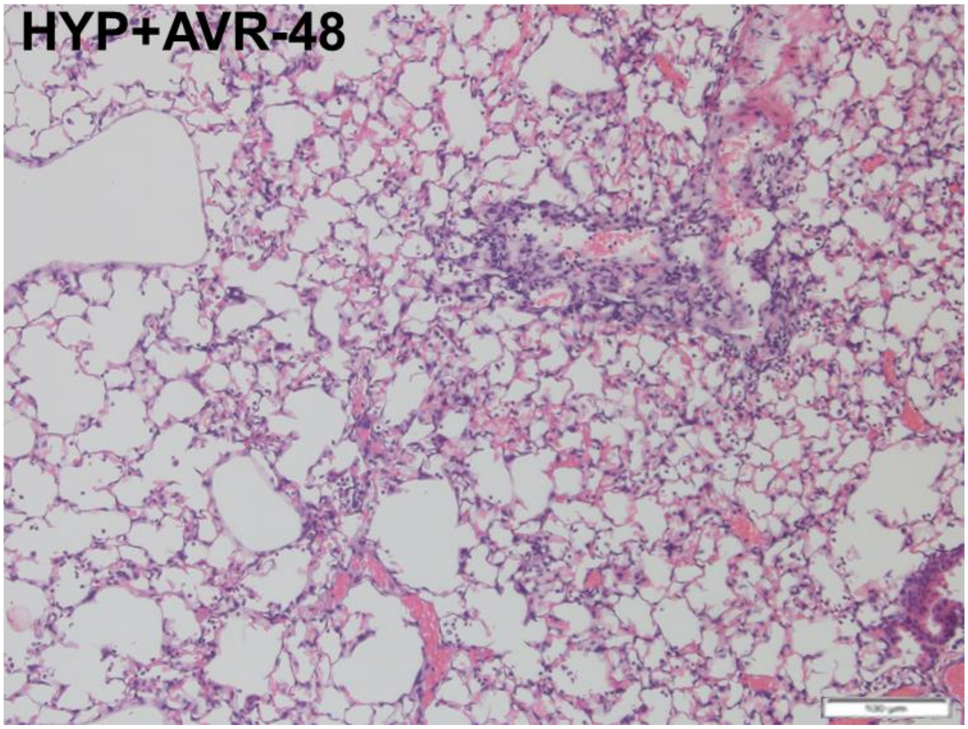

Supplement: Supplementary file 1 [file ijms-22-02573-s001.pdf]
